# New Trends in Melanoma Detection Using Neural Networks: A Systematic Review

**DOI:** 10.3390/s22020496

**Published:** 2022-01-10

**Authors:** Dan Popescu, Mohamed El-Khatib, Hassan El-Khatib, Loretta Ichim

**Affiliations:** Faculty of Automatic Control and Computers, University Politehnica of Bucharest, 060042 Bucharest, Romania; mohamed.el@stud.acs.upb.ro (M.E.-K.); hassan.el_khatib@stud.fim.upb.ro (H.E.-K.); loretta.ichim@upb.ro (L.I.)

**Keywords:** skin lesion, image processing, machine learning, deep learning, neural networks, image classifiers, image segmentation, melanoma detection, statistic performances, review

## Abstract

Due to its increasing incidence, skin cancer, and especially melanoma, is a serious health disease today. The high mortality rate associated with melanoma makes it necessary to detect the early stages to be treated urgently and properly. This is the reason why many researchers in this domain wanted to obtain accurate computer-aided diagnosis systems to assist in the early detection and diagnosis of such diseases. The paper presents a systematic review of recent advances in an area of increased interest for cancer prediction, with a focus on a comparative perspective of melanoma detection using artificial intelligence, especially neural network-based systems. Such structures can be considered intelligent support systems for dermatologists. Theoretical and applied contributions were investigated in the new development trends of multiple neural network architecture, based on decision fusion. The most representative articles covering the area of melanoma detection based on neural networks, published in journals and impact conferences, were investigated between 2015 and 2021, focusing on the interval 2018–2021 as new trends. Additionally presented are the main databases and trends in their use in teaching neural networks to detect melanomas. Finally, a research agenda was highlighted to advance the field towards the new trends.

## 1. Introduction

Melanoma (Me) is known as the deadliest type of skin cancer [1], the incidence of its occurrence increasing for both men and women worldwide every year [2,3]. According to Sun X. et al. [4] the main cause of Me occurrence is exposure to ultraviolet radiation. Due to this excessive exposure, some mutations that occur at the level of melanocytes can lead to Me genesis. Even though it is one of the deadliest types of skin cancers, many studies showed that early detection of Me leads to its treatment in 90% of cases [5]. Currently, the standard method of Me diagnosis is visual analysis by a specialist. However, this method can be time-consuming. Moreover, it can lead to misdiagnosis due to the complexity of providing the diagnosis. The following aspects need to be considered: the number of parameters that need to be analyzed (color, shape, texture, edge, asymmetry, etc.), the fatigue, and the lack of experience of the specialist [6,7,8]. In most cases, the dermoscopic images are acquired and analyzed by the dermatologist, thus achieving a maximum of 84% examination accuracy (ACC) [9,10], which is insufficient. Therefore, the help of a computer-aided diagnosis (CAD) system for Me diagnosis from images is more than necessary [11].

**Figure 1:**
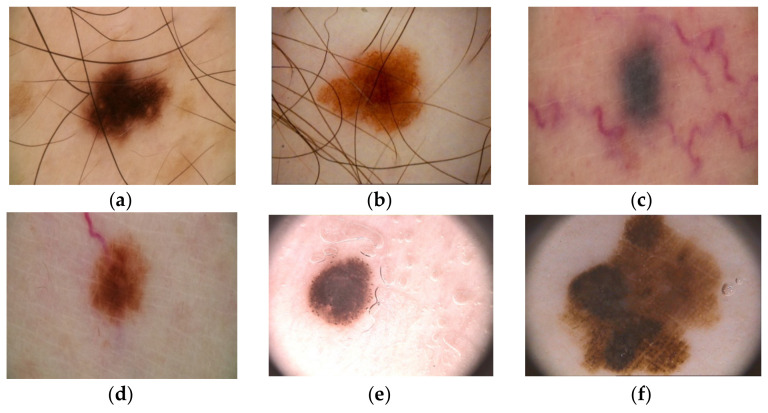
Artifacts in Me images collected from the ISIC 2016 dataset [14]: (**a**–**c**)—presence of hair, (**d**)—presence of blood vessels, (**e**,**f**)—presence of oil drops.

Over time, a lot of researchers have put their ideas together to try to develop an automatic Me detection system based on machine learning (ML) that provides a quick result with high ACC, even if the complexity of skin lesion (SL) images analysis presented many problems [12,13]. In reality, it is a rather complex task to find a suitable diagnosis algorithm due to the presence of artifacts, such as the presence of hair around or even in the lesion, different lesion dimensions, color and shapes, the presence of blood vessels, and other artifacts [14], as seen in Figure 1.

**Figure 2:**
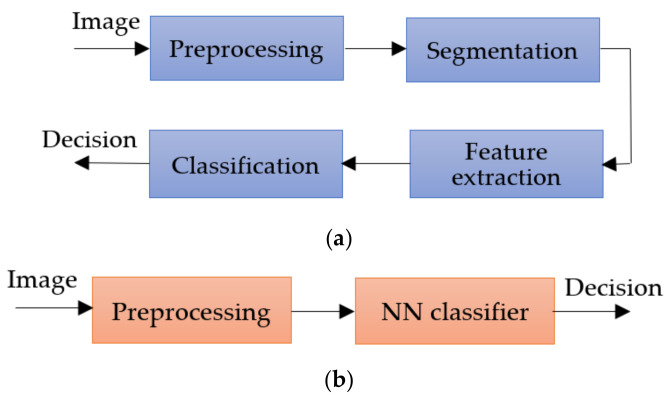
Methods workflow for Me detection: (**a**) classical method, (**b**) NN approach.

The inconveniences caused by these factors led the authors to expand their research a lot but, in principle, most approaches use the same classical method in which the first step is the preprocessing step, followed by segmentation, feature extraction, and then the classification step. The main workflow of the classical method is as shown in Figure 2.

The preprocessing step consists of applying primary operations such as the following: noise removal, data augmentation, resizing, brightness grayscale transformation or brightness corrections, binarization, and, mainly, intensity and contrast enhancement [15]. As the Me images have a high variability of content, the segmentation step is a much-debated topic and a difficult task. This step represents the part of the algorithm that makes possible the image splitting into several sets of pixels [16], with the extraction of regions of interest (RoI) by an automatic or semiautomatic process as the end result [17]. Among the most commonly used techniques for Me detection and segmentation are artificial neural network-based methods (NNs). Considering the variability of Me images, the first-mentioned method (Figure 2a) cannot provide the best results. After the segmentation, the feature extraction step is usually applied. This task consists of reducing the dimensions of the data representation such that this becomes more administrable. Thus, data processing becomes faster and easier, without losing important information. Even so, it is known as a large consumer of resources due to the high number of variables. Generally, if the feature extraction is well done, the detection ACC will increase significantly [16]. In the past, most authors [18,19,20] used the ABCD (Asymmetry, Border, Color, Differential structure) rule as a feature extraction-based method for Me detection, while presently others use deep learning (DL) techniques to make the feature extraction better. The last, and the most discussed step in our review, is the classification step. The goal of this step is to assign a class to an RoI from an image. Manual classification is hard and time-consuming and therefore the interest for developing an accurate automatic classification algorithm increased in last years.

Nowadays, whether it is about segmentation, feature extraction, or classification, the tendency is to use the benefits of Artificial Intelligence (AI) using NN and DL techniques to obtain more accurate results. The main goal of AI is the reproduction of human intelligence, with applications in domains such as autonomous vehicles, search engines, art creation, or medical diagnosis. In the case of Me detection by applying AI, promising results were obtained, reaching a level where only visual inspection of SL is no longer a reliable solution. Known as a subset of the AI, the classical ML algorithms were proposed first as a solution for automatic Me detection. Mainly, ML uses the previous experience to improve the given results [21]. The system first extracts the needed features to create the training data. After the training data are obtained, supervised or unsupervised learning is used in the learning process. Generally, most papers used the supervised learning models, being more accurate. As has been observed also in other areas in which it is applied, the classical ML-based methods showed promising results, but also some limitations. For example, a large amount of data are needed to train the system, the learning phase takes a long time, and ML presents a high error-susceptibility. Thus, the authors turned their attention to NN and DL techniques.

NNs consist of a collection of neurons that simulates the function of neurons in a human being. In such a network, the neurons are connected to each other, each connection being assigned a weight, helping the neurons to give the necessary output. The authors prefer the NNs because they present benefits, such as distributed memory, the possibility of giving good results with a small amount of information, or the possibility of parallel processing. For training, the system error is calculated by taking the difference between the predicted value and the output target. Using this calculated error, the system adjusts its weights until the error is minimized.

Most Me detection papers used the feedforward and the recurrent NNs to obtain a high ACC result. Better results were obtained by the authors by using DL models such as CNN or Recurrent NN. The CNNs are NNs with at least one convolution layer. At present, different applications including Me detection systems obtain the best results.

The main aim of this work is the analysis of new trends of approaches used in the automatic SL detection field (especially Me). The paper focuses on presenting the growth trend of using NN techniques when developing such a system. The rest of the paper is organized as follows. Section 2, named Materials and Methods, presents the search strategy for motivation and selection of the recent relevant papers to establish the new trends in the Me detection by NN. Section 3 addresses the main DSs used in the selected articles, focusing on public DSs. The most important NNs used today for Me detection, classification, and segmentation are described and analyzed in Section 4. Section 5 presents the new directions of NN implementation in Me detection, taking into consideration individual NNs, multiple NN configurations based on decision fusion, and hybrid configurations consisting of NNs and other intelligent classifiers. Finally, a Discussion section (Section 6) compares the results of this paper with other similar review/survey papers highlighting the novelties.

## 2. Materials and Methods

**Figure 3:**
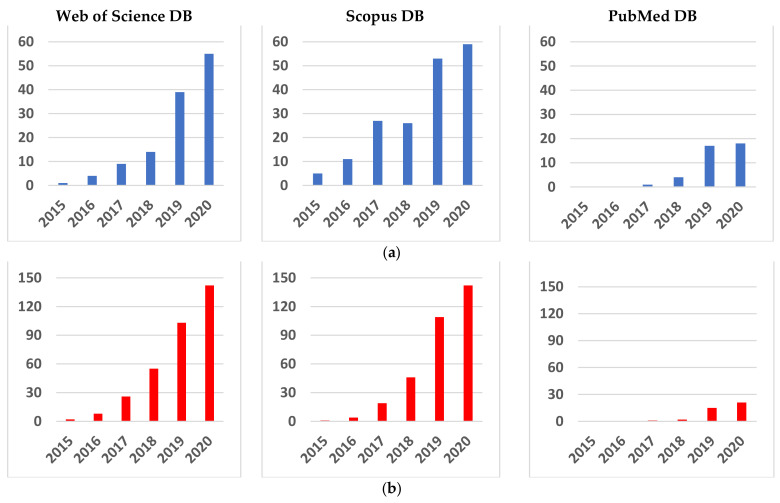

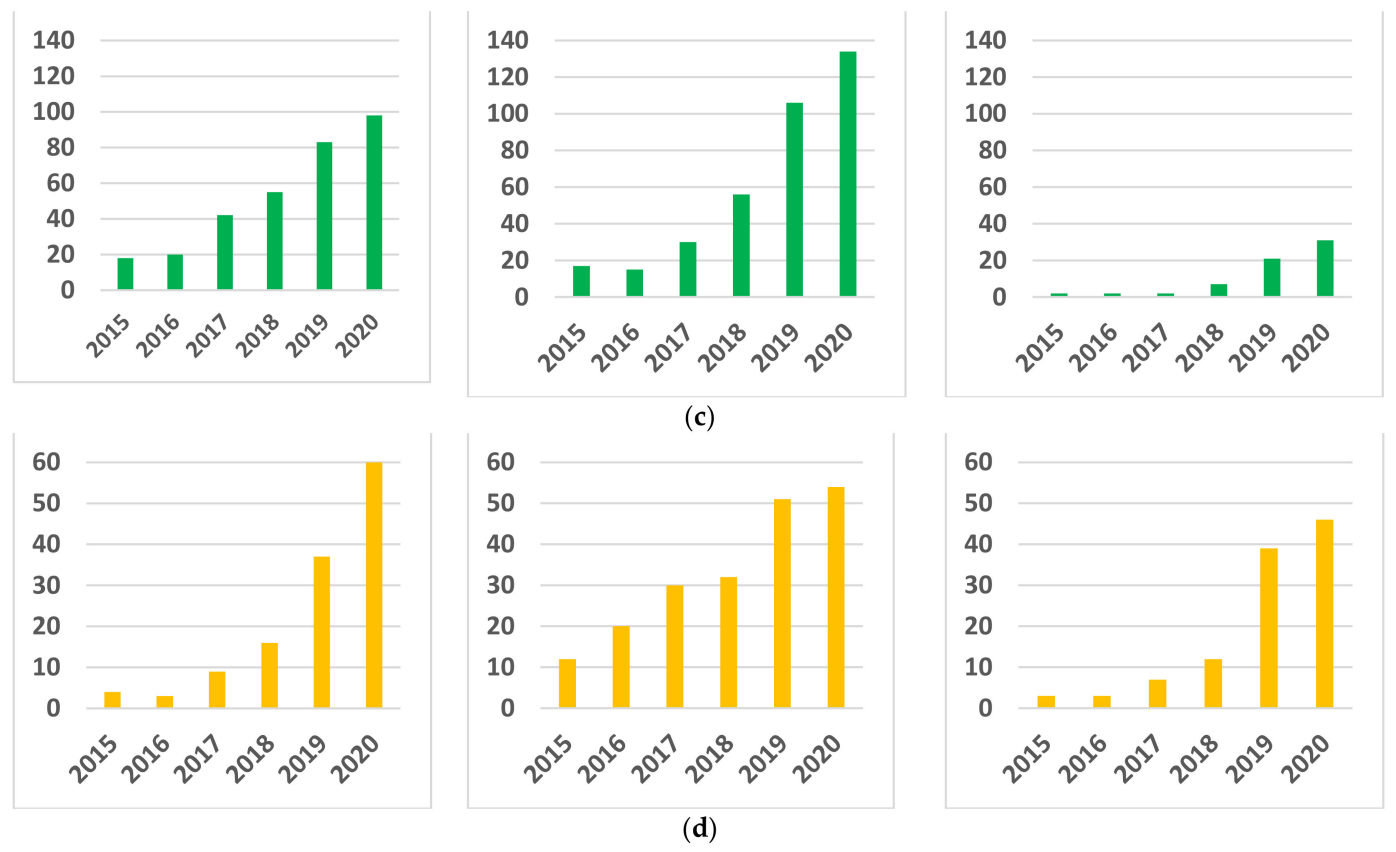
Searches for important terms in the Web of Science, Scopus, and PubMed DBs between 2015 and 2021 with the AND connector: (**a**) CNN AND Me, (**b**) DL AND Me, (**c**) ML AND Me, and (**d**) AI AND Me.

Although the papers that addressed Me detection and NN use separately are older and their research is well-established, the study of Me detection by NN algorithms is relatively recent (Figure 3a). As we considered the new trends in Me detection using NNs, we searched the following DSs: Web of Science, Scopus, and PubMed between 2015 and 2021 considering the following topics: melanoma, skin lesions, artificial intelligence, machine learning, deep learning, and convolutional neural networks. The search was split between combinations of keywords using the “AND” connector: CNN AND Me (Figure 3a), DL AND Me (Figure 3b), ML AND Me (Figure 3c), and AI AND Me (Figure 3d). It can be observed that the increase in research is exponential in the cases of CNN AND Me, DL AND Me, and AI AND Me and quasilinear in the case of ML AND Me. The number of publications identified according to the search in the database is labeled on the y-axis in Figure 3.

**Figure 4:**
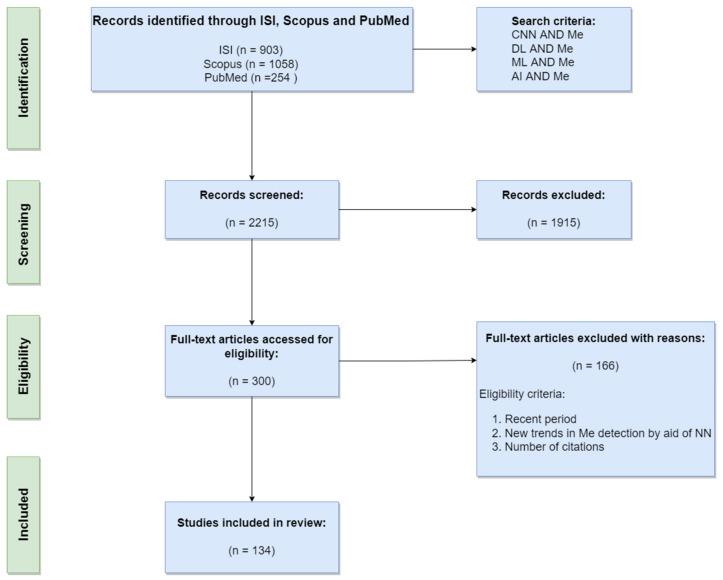
PRISMA flow diagram of our research.

As many as 300 full-text papers were analyzed from Web of Science, Scopus, and PubMed, of which we selected 134 research papers for this review. The main criteria for paper selection were: the recent period, new trends in Me detection by the aid of NN, visibility, and impact of contributions (publishing in high-rank conferences and journals, number of citations). The most representative articles covering melanoma detection based on neural networks, published in journals and impact conferences, were investigated between 2015 and 2021 (92% of references), focusing on the interval 2018–2021 (80% of references) as a recent period. In terms of new trends of using NNs for detection, segmentation, and classification of Me, we noticed the following directions: systems using one single CNN most often modified and adapted for Me, systems using multiple CNNs, and systems using CNN combined with other classifiers. Details will be given in Section 5. Although the number of citations is relative, in general for older papers it is higher than for new ones (2021). However, obviously, there are exceptions. Due to this, we did not set a threshold for the number of citations. We had in mind that most papers follow what we have stated as new trends and obviously have a reasonable number of citations. The high-rank of the journal refers to Category Quartile Q1, Q2, and the Journal Impact Factor greater than 2.2 in Web of Science 2020. About 50% of the total references meet this criterion. For the systematic review and meta-analysis, we used a PRISMA (Preferred Reporting Items for Systematic Reviews and Meta-Analyses) flow diagram (Figure 4).

Most relevant papers concerning the aspects of new trends in the last period (related to Me, DSs, NNs, decision fusion, and combined networks) are detailed in Section 3, Section 4 and Section 5. To compare every analyzed paper, the important statistical performances are presented. The performance evaluation metrics most used in SL detection, segmentation, and classification are the following: Accuracy, Precision, Sensitivity, Specificity, F1-score, and Jaccard index. The formulas are listed in Table 1, where TP is true positive, TN—true negative, FP—false positive, and FN—false negative cases. The emphasis was on accuracy (ACC), F1 score (F1—Dice Coefficient), and Jaccard index (IoU—Intersection over Union).

## 3. Datasets Used in Melanoma Detection

The systems presented in this study are based on AI, which means that they are meant to learn from one or more DSs (both small and large ones). The DSs were built in collaboration with doctors/medical specialists. These DSs are composed of high-quality, well-selected images, previously analyzed, labeled, and potentially segmented by medical specialists from the respective domain. Our study aims to present the growth trend of such automated systems able to diagnose, segment, or detect certain SLs (especially Me) based on existing papers in the literature. The outcome of these papers was possible because of some existing public DSs. In this section, we will present some of the popular DSs which were used in a lot of papers from the SL domain. Among these DSs, we can find PH2, ISIC 2016, 2017, 2018, 2019 challenge DSs, HAM10000, DermNet Atlas, Dermatology Atlas, DermIs, and MED-NODE (Table 2).

One of the most used dermoscopic databases (DB) in certain papers is PH2. As specified in [22], this DB was built in Portugal at Hospital Pedro Hispano as a collaboration between multiple medical entities. The images from this DB contain a total number of 200 dermoscopic images (80 common nevi, 80 atypical, and 40 Me). The images are 8-bit RGB color images with a resolution of 768 × 560 pixels, carefully selected by taking into consideration the quality, resolution, and dermoscopic features. For each image in the DB, the manual segmentation and the clinical diagnosis of the SL as well as the identification of other important dermoscopic criteria are available.

Other important DSs used in this area are provided by ISIC (International Skin Imaging Collaborative) which provides expertly annotated DSs containing digital SL images of different versions (2016, 2017, 2018, 2019, and 2020) to facilitate CAD of multiple SL diseases [23,24]. These DSs were used at the International Symposia in Biomedical Imaging (ISBI).

ISIC 2016 DS [14] contains 900 dermoscopic lesion images in JPEG format, with EXIF data stripped as training data and 379 images with the same format as testing data. The images from this DS have a resolution between 576 × 768 and 2848 × 4288, which means that, in some cases, resizing operations might be needed.

ISIC 2017 [25] contains a total number of 2750 SLs where 2150 can be used as training data and 600 can be used for testing data. The resolutions of these images are between 540 × 722 and 4499 × 6748. Like in the previous DS, in some cases, resizing operations might be needed.

The ISIC 2018 challenge DS [25] was used for Skin Lesion Analysis towards the Melanoma Detection challenge [26]. The DS is quite large (about 10.4 GB), and it contains 2594 images and 12,970 corresponding ground truth response masks (5 for each image) as training data and 1000 images (about 2.2 GB) as testing data. The SL are RGB images in JPG format and the masks are grayscale images in the PNG format [27]. The ISIC 2018 challenge was composed of three challenge tasks. Within the first two tasks, the participants were using 2594 images already presented, while, within the last task, representing a classification task, the participants used HAM10000 DS, which of course is another very popular DS, publicly available through ISIC archives. HAM10000 is composed of 10,015 images out of which 1113 are Me. All images in the DS are in JPEG format (8-bit color depth) and were all manually cropped with the lesion centered to 800 × 600 px at 72DPI and manual histogram corrections applied to enhance visual contrast and color reproduction [28]. ISIC 2019 and ISIC 2020 are new variants of ISIC DSs with more and more images in comparison with previous ones [23].

Another popular DS used in skin cancer detection systems is MED-NODE DS, which contains 70 Me and 100 nevus images from the digital image archive of the department of dermatology, University Medical Center Groningen [29].

Dermofit image library is a DS, property of the University of Edinburgh, which can be used only in medical imaging research. The DS is composed of 1300 high-quality SL images and contains ten different classes including Me (76), Melanocytic Nevus/Mole (331), Seborrhoeic Keratosis (257), Basal Cell Carcinomas (239), etc. [30]. Each image in this DS is a normal RGB captured with a quality SLR camera under controller (ring flash) indoor lighting. The images were labeled based on expert opinion (dermatologists and dermatopathologists) and binary segmentation masks, marking the lesions themselves, are also included. To access this DS, there is a need for a one-time purchase-only license.

DermNet Skin Disease Atlas is another DS used in research related to skin lesions detection, segmentation, and classification problems. This DS is composed of over 22,000 images (only 21,844 found as relevant) divided into 23 types of skin diseases (superclasses) [31]. The images are of the RGB type in JPEG format and the resolutions vary from image to image [32].

DermIS is also a publicly available dermoscopic image DS, widely used in the literature for SL detection, segmentation, and classification purposes and it is composed of a total number of 300 Me images [33]. The DS is available on [34] and provides the ability to search for dermoscopic images by category (face, hands, legs, etc.).

Another popular DS used for SL detection, segmentation, and classification was Dermquest which is an online medical atlas for dermatologists and dermatologist-based healthcare professionals [35]. The DS was publicly available (it is not currently) and contained over 22,000 clinical images.

Table 2 gives a summary of the properties of the DB/DS used in the studied references and illustrates the availability of the discussed and primarily used DSs identified in our study/survey.

**Figure 5:**
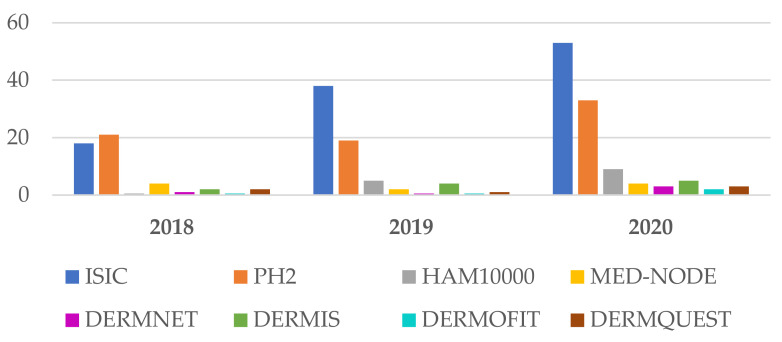
Frequently DSs used in Me detection between 2018 and 2020.

**Figure 6:**
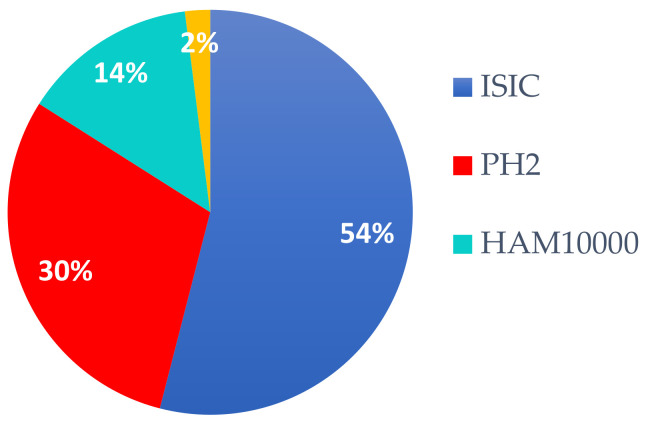
The four most used DSs for Me detection in 2021 (percentage).

The ISIC archives, PH2, HAM10000, MED-NODE, DermIS, and Dermquest DSs are free and publicly available for SL diagnosis research. Watermarks usually mean noise in the images when it comes to DL systems which are oriented towards learning different patterns. Therefore, researchers willing to access the high-quality images without watermarks from DermNet will need to purchase a license. This is one of the reasons we observed that DermNet is not widely used (it can be seen in Figure 5 and Figure 6), even if it is a large data set where the non-watermarked, high-quality images might make the difference in the DL process.

According to our study, as can be seen in Figure 5 (focusing on the period 2018–2020, as new trends) and Figure 5 (for 2021), the most widely used DSs in SL diagnosis research are the ones included in ISIC archives (containing also H10000). The first reason is that these DSs are quite consistent and very well labeled by domain experts, and the second reason might be the annual challenges posed by consistent prices. The second place is occupied by PH2, which is a small DB but, according to our study, the trend is to use small DSs for system/solution validation and to use large DSs for learning such as with DL and TL (transfer learning) systems. As can be seen in Section 4, data augmentation is frequently used. For the year 2021, a separate evaluation (based on percentage) is presented in Figure 6. It can be observed that the trend is maintained (54% ISIC and 30% PH2).

## 4. Neural Networks Used in Melanoma Detection, Segmentation, and Classification

According to the current study related to SL detection, segmentation, and classification papers in the literature, it turned out that the majority of these kinds of tasks used NNs, CNNs, DCNNs (Deep Convolutional Neural Networks), and TL for NNs. It can be observed that the trend throughout the years, in general, and not strictly related to SL diagnosis systems, is that researchers used to design deep networks with a lot of hidden layers (either convolutional or fully connected layers) to obtain better results. It is normal that, when this happened at first, the time complexity for training, classification, detection, or segmentation was somehow neglected, all works being more focused on better statistical performance (required by diagnostic specialists). As a consequence, the majority of works related to Me detection, segmentation and/or classification systems are based on NNs. Table 3 illustrates the most used NNs in such applications. As we are mostly interested in the usage trend of NNs used in Me diagnosis, this section presents the architecture of the basic NNs widely used in these kinds of applications.

**Figure 7:**
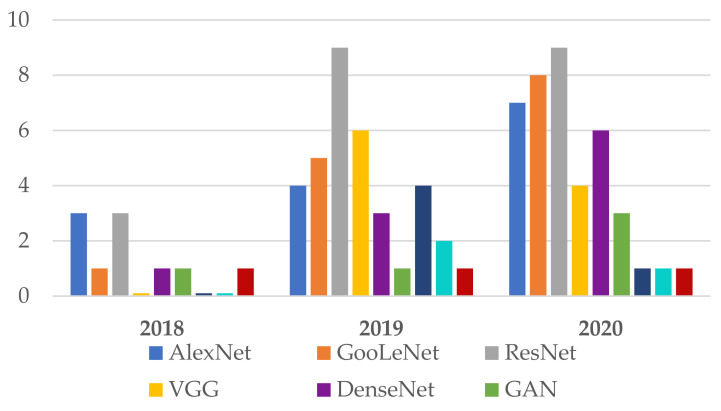
Frequently NNs used in Me detection between 2018 and 2020.

**Figure 8:**
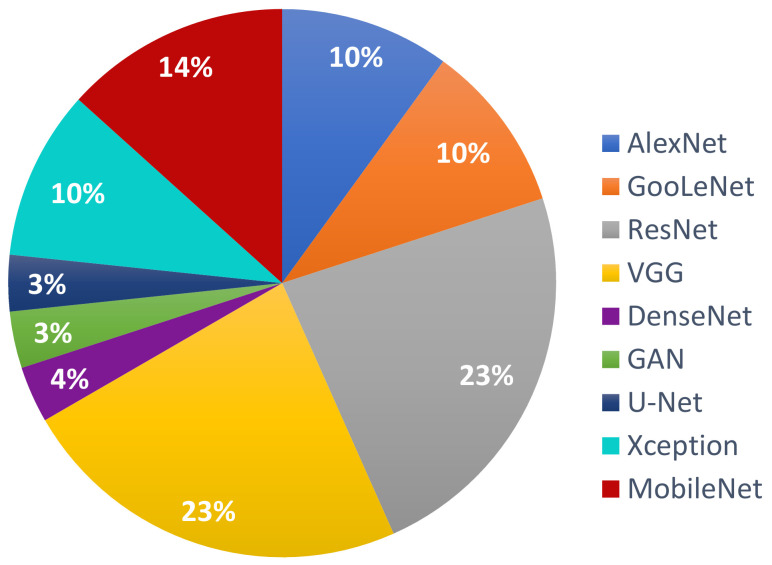
The most used NNs for Me detection in 2021 (percentage).

Following the investigation of the Web of Science DB between the years 2018 and 2020 (Figure 7), it can be found that the most used NN in the detection of Me were those in the family ResNet, followed by the families: VGG, GoogLeNet, and AlexNet. For the year 2021, the tendency is for ResNet and VGG networks (Figure 8). Figure 7 marks the number of appearances in the years 2018, 2019, and 2020, and Figure 8 the percentage of appearances in 2021 (unfinished year).

### 4.1. AlexNet

**Figure 9:**
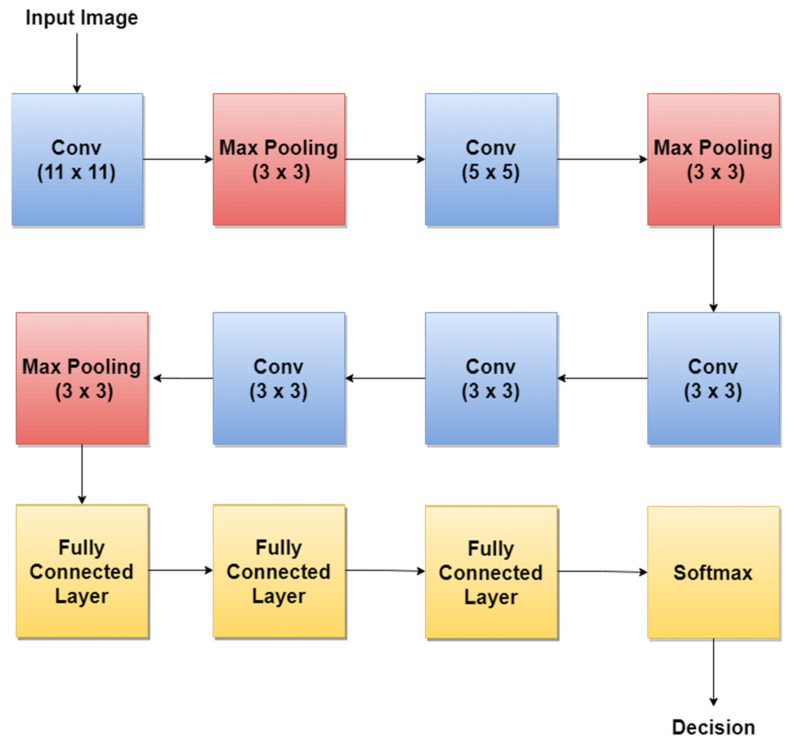
AlexNet basic architecture.

AlexNet [93] is one of the first CNNs widely used in SL classification tasks via TL. The basic architecture (Figure 9) is composed of eight layers, out of which five are convolutional layers (Conv) and three are fully connected layers (FC). The first and second layers are followed by Max Pooling layers (MPX) and Local Response Normalization (LRN), while the third, fourth, and fifth are followed by ReLU (Rectified Linear Units) [94]. The last layer (Softmax layer) has 1000 neurons and is used for the classification task (1000 classes). The number of layers specified in the above architecture is not what makes AlexNet special. AlexNet replaced the Tanh function with ReLU for speed enhancement in terms of training time. In Figure 9, at each layer, the number of neurons is specified.

For example, in 2018, the authors in [45] trained AlexNet using TL, together with three other architectures: GoogLeNet, ResNet, and VGGNet to achieve a better ACC in such classification tasks. By training AlexNet to classify SL, the authors obtained an average ACC of about 85%. Other research papers such as: [12,73,74] used the trained AlexNet for SL diagnosis.

### 4.2. GoogLeNet/Inception

**Figure 10:**
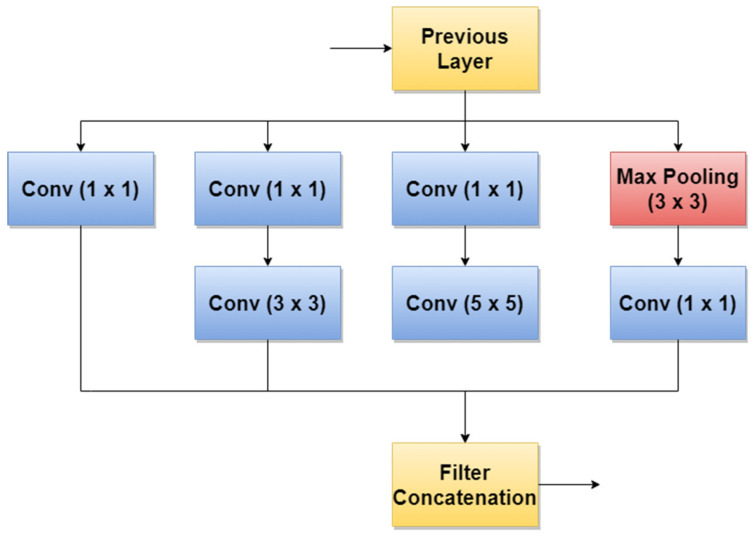
Inception module used in GoogLeNet.

GoogLeNet, also named Inception v1, is a CNN proposed by researchers at Google in 2014 [95]. Its architecture was the winner of the ILSVRC 2014 image classification challenge (ImageNet Large Scale Visual Recognition Challenge 2014) and performed better in terms of error rate compared with previous winners: AlexNet in 2012 and ZG-Net in 2013. New features of GoogLeNet are the following: 1 × 1 convolution, global average pooling, an Inception module, and an auxiliary classifier for training. The 1 × 1 convolution blocks were introduced to decrease the number of parameters in general (weights and biases), which of course led to a depth increase of the architecture. The network’s basic block is the Inception module, where 1 × 1, 3 × 3, 5 × 5 convolutions, and 3 × 3 Max Pooling blocks perform in parallel. The outputs of these blocks are concatenated and fed to the next layer. The Inception module was introduced since different convolutions blocks of different sizes handle objects better at multiple scales. Figure 10 illustrates the components of the Inception module used in GoogLeNet.

**Figure 11:**
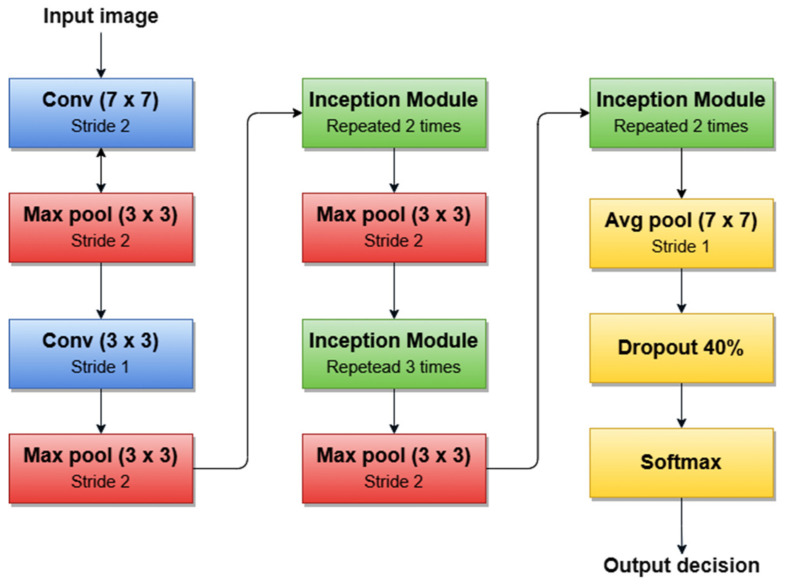
GoogleNet architecture’s simplified block diagram.

A simplified architecture of GoogLeNet is 22 layers deep (Figure 11). The network takes a color image (RGB) of size 224 × 224 pixels as input and provides the classification result (out of 1000 classes) as output, using a Softmax layer of 1000 neurons. Another important aspect to mention is that all convolutions inside the architecture use ReLU as an activation function.

**Figure 12:**
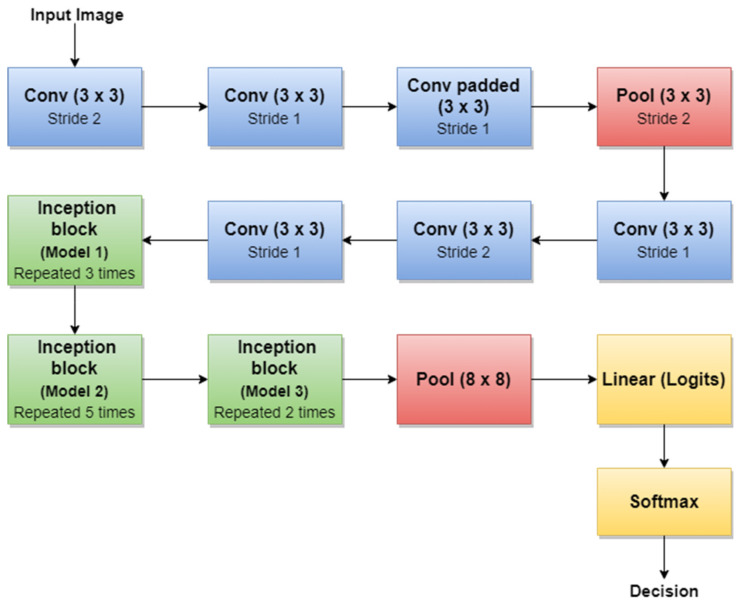
Inception v3 basic architecture.

For example, the authors in [45] used the first version of GoogLeNet (Inception v1) as the basic CNN from which they started TL for SL diagnosis. Additionally, the authors in [5] (published in 2020) trained Goog-LeNet for the Me classification task, which shows that this architecture added a lot of value with its newly introduced features. Recently, a series of published SL diagnosis systems used newer versions of GoogLeNet. For instance [43] related to Me and the nevus SL classification task uses the Inception v3 NN [96] with 42 layers deep. Figure 12 illustrates the overall architecture of the Inception v3 network.

An important observation is that Batch Norm (Batch Normalization) and ReLU blocks are used after each convolution. The basic idea of Inception v3 NN and what makes it more special than the first version (GoogLeNet—Inception v1) is to reduce the number of connections/parameters without decreasing the network efficiency. This is one of the reasons why researchers also investigate the performance of this CNN in their applications. Inception v3 uses “Factorizing convolutions” by replacing the 5 × 5 convolution filter represented in Figure 10 with two convolution filters 3 × 3. This procedure reduces the number of parameters from 25 to 18. The same technique was also used in VGG Net [97]. Another important novelty introduced by Inception v3 is related to factorization into asymmetric convolutions which means that a 3 × 3 convolution filter will be replaced by one 3 × 1 convolution filter followed by one 1 × 3 convolution filter.

### 4.3. VGG Networks

**Figure 13:**
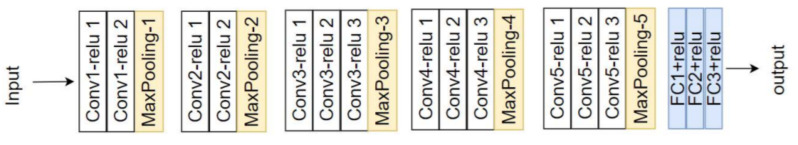
VGG 16 network architecture [98].

VGG is a NN family with the first representative VGG 16, which is widely used in SL diagnosis. VGG16 [98] is slightly similar to, but larger, than AlexNet, being 16 layers deep and containing only small 3 × 3 convolution filters (Figure 13). For instance, the authors in [45,47,53] used a TL technique to train VGG 16 to achieve SL diagnosis.

VGG 16 model achieves a 92.7% top-5 test ACC in ImageNet BD (14 million images belonging to 1000 classes) and was the winner of ILSVRC-2014. With this model, an improvement can be seen over AlexNet, since it replaces large filters such as 11 × 11 and 5 × 5 with multiple smaller 3 × 3 filters, making the network deeper (ascending trend for obtaining a better ACC). The same behavior of “Factorizing Convolutions” was also used in GoogLeNet Inception v3.

**Figure 14:**
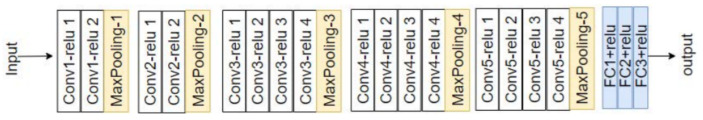
VGG 19 network architecture [98].

VGG 19, shown in Figure 14 [98], is another VGG network used in SL (especially Me) diagnostic research papers in the literature. This time, the model becomes deeper (19 layers, out of which 3 are fully connected layers). According to our survey, examples of paper works related to SL diagnosis are [43,47]. Both papers mentioned as examples were published in 2020 and represent comparative studies between multiple networks to find the most accurate and precise ones for SL diagnosis tasks. What can also be noticed is that, in terms of compared networks, apart from VGG 16 and VGG 19, other deeper networks such as ResNet-50 (50 layers deep) and DenseNet-201 (201 layers deep) are involved. This means that the trend in using NNs for SLs diagnosis is to use deeper networks to achieve better ACC and precision. Of course, this can lead to more and more network parameters and large computation time in terms of the learning task, which will continue to be a subject of research.

### 4.4. ResNet

**Figure 15:**
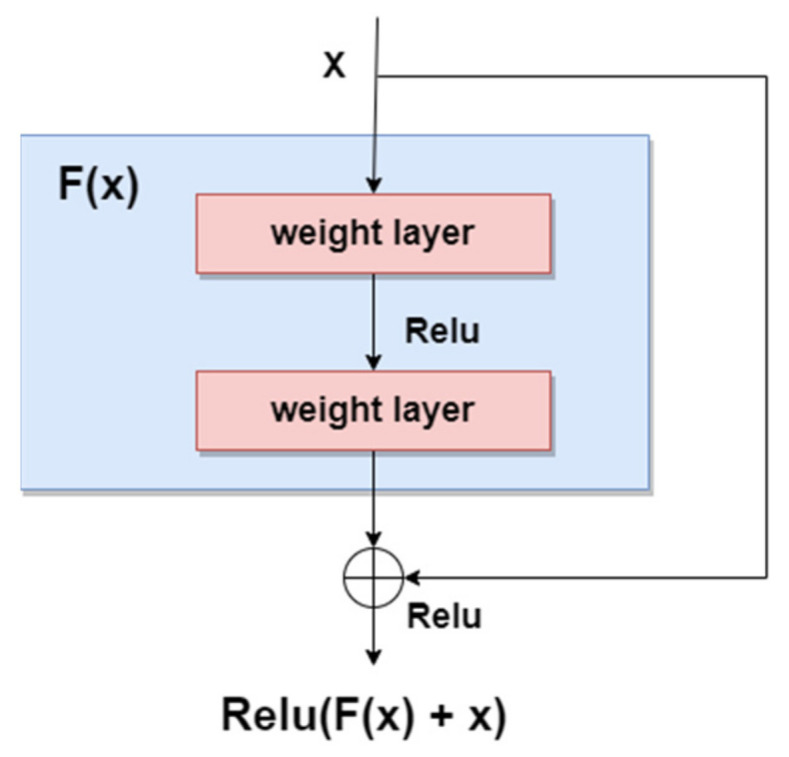
Residual block.

As we mentioned in the previous sections, the general trend for segmentation, detection, and classification tasks is to use deeper NNs. However, it was demonstrated that, as we go deeper with more and more layers with “plain” networks, the training error will start to increase over time. Therefore, very deep NNs are in general hard to train because of vanishing and exploding gradients kind of problems. To avoid this issue, researchers introduced “skip connections” in the networks which allow them to take the activation from one layer and feed it to another layer, even much deeper in the NN. This allows building “Residual” networks, instead of “Plain” networks, thus building very deep NNs (over hundreds of layers deep). The newly introduced “Residual” network [99] solves the problem of the vanishing gradient in deep NNs by allowing the shortcut presented in Figure 15. In this way, the gradient can flow through. With this new feature, ResNet won first place in the ILSVRC 2015 competition with an error rate of 3.57%. It also won the COCO 2015 competition for detection and segmentation problems.

**Figure 16:**
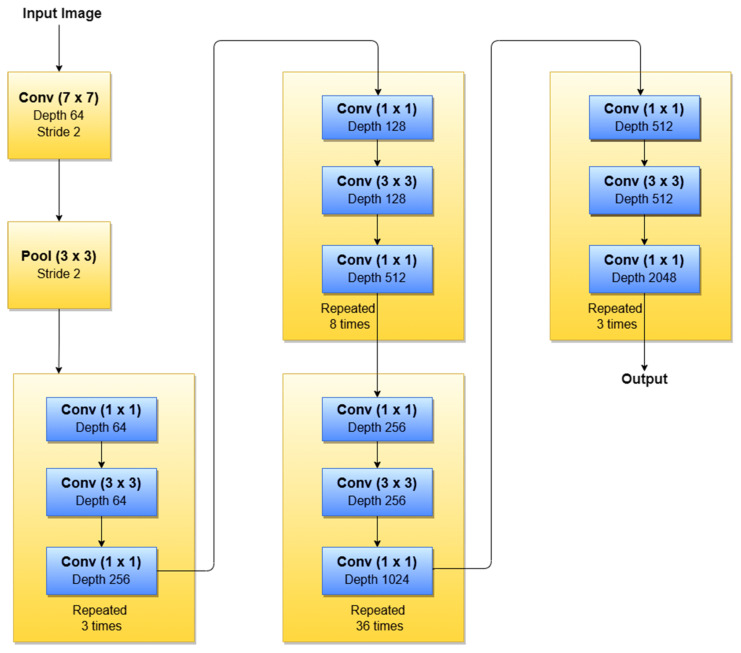
ResNet-152 basic architecture.

According to our search related to SLs diagnosis (Table 3), in terms of the ResNet family, the most used NNs for detection, segmentation, and classification task, are ResNet-34, ResNet-50, ResNet-101, and ResNet-152. As can be seen in Figure 16, ResNet-152 is a 152-layer-deep CNN composed of residual blocks which solve the vanishing gradient issue when training deep NNs. An example of an SL diagnosis paper that uses ResNet-34 is [47]. Another residual network used in SL diagnosis tasks is ResNet-50 (50 layers deep), which was used for instance in [41,43,47,50], all published in 2020. A residual network 101 layers deep, used in SL diagnosis, is ResNet-101 ([5,42,43,50], all published in 2020). Of course, there are also other studies, such as [38,39,48,96], that use a deeper “residual” network (representing the trend of using more deeper networks for better ACC) called ResNet-152 (152 layers deep).

### 4.5. YOLO Networks

**Figure 17:**
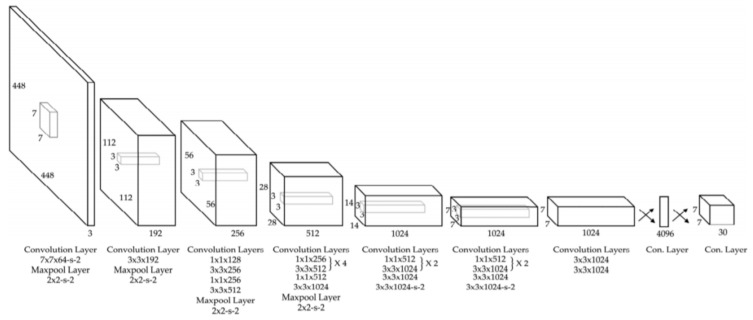
YOLO v3 architecture [101].

YOLO (You Only Look Once) is a CNN widely used in real-time object detection tasks and commonly used network in Me detection papers (usually YOLO v3 and YOLO v4). According to [100], YOLO is a “new approach to object detection” by using a single NN to “predict bounding boxes and class probabilities directly from full images in one evaluation”. YOLO is composed of 24 convolutional layers followed by two fully connected layers which were pre-trained on ImageNet DB, similarly to other commonly used networks. As can be seen in Figure 17 [101], the network contains some alternating 1 × 1 convolution filters which are mainly used to reduce the features space from the preceding layers. This looks similar to what GoogLeNe—Inception v3 introduced. There are multiple versions of YOLO, out of which, according to our research, the most used CNNs for Me detection tasks are YOLO v3 and YOLO v4 [88].

YOLO v3 is an incremental improvement of the previous YOLO v2 which was based on DarkNet-19 network. According to the authors in [102], the network is bigger than YOLO v2, with increased ACC, and is fast enough. The authors proposed a hybrid approach between DarkNet-19 and a residual network (inspired from ResNet). The new architecture is based on 53 convolutional layers called DarkNet-53. As we already mentioned, YOLO v3 is used in Me detection tasks. For instance, the authors in [89,90] used YOLO v3 for benign/malignant Me or seborrheic keratosis detection. There is also the YOLO v4 version with an increasing speed, used in Me detection and segmentation [88].

### 4.6. Xception Network

**Figure 18:**
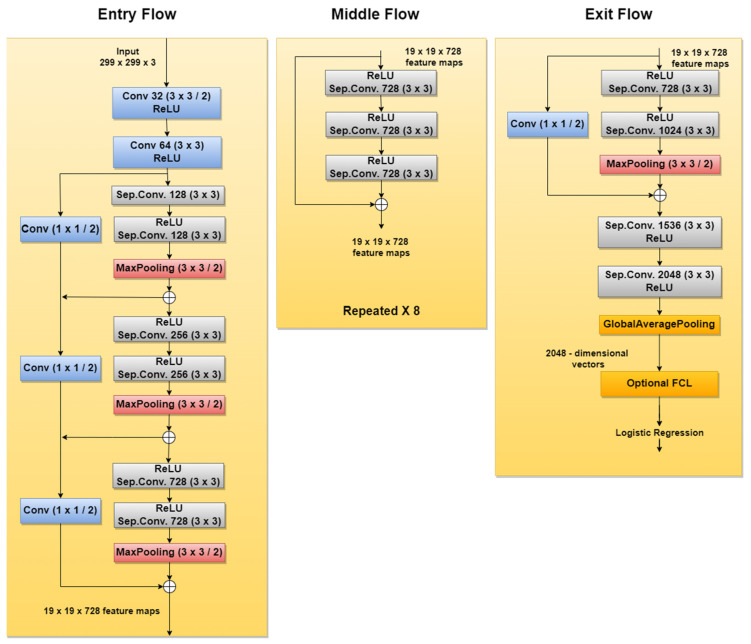
Xception network architecture.

Xception is another CNN used in SL diagnosis tasks. For instance, new related papers are [42,43], both being published in 2020. According to [103], this network was inspired by GoogLeNet Inception NN also developed by Google researchers and was meant to obtain better performance by replacing the standard Inception modules with depthwise separable convolutions. The Xception architecture (Figure 18), which outperforms Inception v3, contains 36 convolutional layers structured in 14 modules, all with residual connections around them [104].

### 4.7. MobileNet

MobileNet is a type of NN designed for mobile and embedded vision applications [105]. Since this CNN is deployed on mobile devices, memory usage should be taken seriously into consideration. Therefore, to decrease the complexity and to reduce the model size, the architecture is based on depthwise separable convolution blocks, as in the case of Xception NN described in the previous section.

There are multiple versions of MobileNet, out of which, according to this research, MobileNet-v1 and MobileNet-v2 are the most used in SL diagnosis papers. For instance [43,47], both published in 2020, use MobileNet-v1; meanwhile, newer papers such as [87] use MobileNet-v2 (deeper and improved version of MobileNet-v1) in such applications.

As we already mentioned, MobileNet NN reduces the complexity and number of network parameters using depthwise separable convolutions (1 × 1 convolution applied on each of the RGB channels). However, it also uses pointwise convolution with a 1 × 1 kernel (depth equal to the number of channels of the image) which iterates through every single point. To this end, MobileNet-v1 uses 13 blocks composed of depthwise separable convolution and pointwise convolution. However, researchers were focused on obtaining better results. Therefore, MobileNet-v2 came about as an improved version of MobileNet-v1. The first important change was marked by the fact that the network is now composed of 17 bottleneck blocks, each of them containing an expansion module, a depthwise separable convolution, and a pointwise convolution. The expansion block was introduced to increase the size of the representation within the bottleneck block to allow the NN to learn a richer function. The pointwise convolution will then “down” project the data so that they reach the initial size. Another important issue introduced in MobileNet-v2 is the residual connections around the bottleneck blocks, to solve the “vanishing gradient” problem, as in the case of ResNet. Of course, both versions end with a Max Pooling layer, followed by Fully Connected layers, and finally followed by a Softmax layer.

### 4.8. EfficientNet

**Figure 19:**
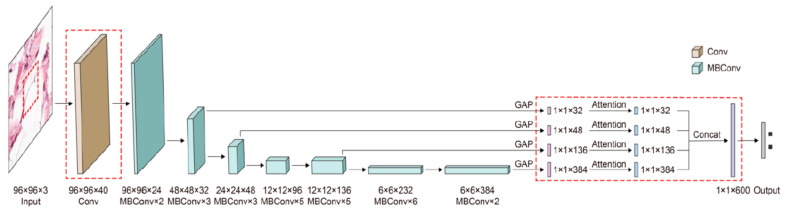
EfficientNet architecture [107].

As we have already mentioned in previous sections, researchers tend to obtain better results in terms of ACC and other performance metrics. For this to happen, the trend is to design deeper CNNs. For example, ResNet can be scaled up to ResNet 200 by increasing the number of layers. Authors in [106] propose a novel model scaling approach that uses compound coefficients to scale up CNNs in a more structured manner. This method uniformly scales each dimension with a fixed set of scaling coefficients. The authors also demonstrated the effectiveness of the proposed method on scaling up MobileNets and ResNets. In the same paper, they also build different versions of EfficientNet (EfficientNet B0–B7), all of them with better ACC than the networks with which they were compared. Another example of a recent paper [47] used EfficientNet to improve ACC for pigmented SL classification. The architecture (Figure 19) is based on MBConv blocks (inverted residual blocks), originally applied on MobileNet-v2 [107].

### 4.9. DenseNet

**Figure 20:**
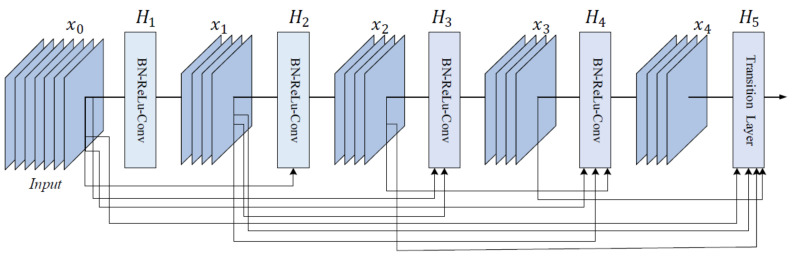
Five-layer DenseNet architecture [108].

DenseNet is a CNN family often used in SL diagnosis. Examples of papers using DenseNet (especially DenseNet-201) are ([1,41,47], all of them published in 2020). Therefore, DenseNet represents a trend for recently published papers because of its efficiency and better ACC. The reason is that, in the initial paper [108], the authors introduced densely connected layers, thus modifying the standard CNN architecture as in Figure 20. In DenseNet, each layer is fed with additional inputs from all preceding layers and provides its own input/feature map to all subsequent layers. In this way, each layer obtains knowledge from previous layers. Therefore, it is obvious that this becomes more powerful than ResNet, obtaining a stronger gradient flow, more diversified features, and a smaller network size. DenseNet-121, DenseNet-169, DenseNet-201, and DenseNet-264 are DenseNet networks presented in different works.

### 4.10. U-Net

**Figure 21:**
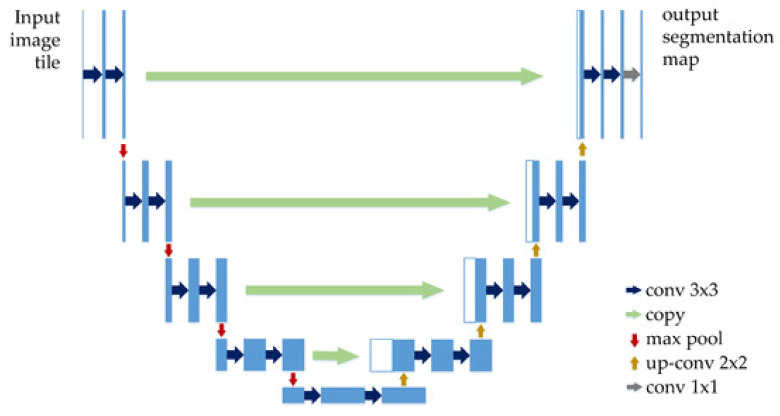
U-Net architecture [110].

Recent papers such as [58] used U-Net CNN for SL segmentation. As can be seen in Figure 20, U-Net has a “U” form, being composed of 23 convolutional layers. After each max pooling operation, the number of feature channels is increased by the previous number of feature channels, multiplied by two. The number of channels is increased until it reaches 1024 and then starts to decrease (dividing by 2, after each 2 × 2 up-conv block). This architecture contains four sections: the encoder, the bottleneck, the decoder, and the skip connections (Figure 21). The bottleneck layer is a section between the down-sampling path (encoder) and up-sampling path (decoder), containing the smallest size of the feature map and the biggest number of filters. The skip connections are between the corresponding blocks of the encoder and decoder.

According to the original paper [109], U-Net achieved very good performance on very different biomedical segmentation applications. This is one of the important reasons why researchers tend to use it in Me detection and segmentation-related papers.

### 4.11. Generative Adversarial Network

**Figure 22:**
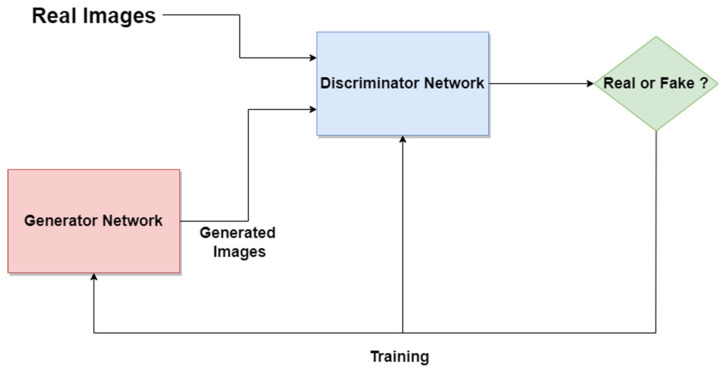
GAN standard network architecture.

The Generative Adversarial Network (GAN) is another type of artificial NN that was used in the design of Me and SL diagnosis and segmentation systems. The GAN is composed of two different networks (main blocks), as can be seen in Figure 22. The first one is the generator network which learns how to generate real-like data, while the second one is a discriminator network which learns how to detect fake data and not to classify them as real data. Both networks are competing and playing an adversarial zero-sum game [111]. The main blocks try to optimize objective functions. GAN was proposed for image synthesis tasks. Starting from this idea, the GAN is used in melanoma segmentation as a generative model based on supervised learning.

According to our research, examples of research papers in this domain are [64,65,66,112], all of them proposing modified variants of GANs, such as SPGGAN (Self-attention Progressive Growing of Generative Adversarial Network), DCGAN (Deep Convolutional Generative Adversarial Network), DDGAN (Deeply Discriminated Generative Adversarial Network), LAPGAN (Laplacian Generative Adversarial Network), etc. There were other research papers involving the combination of GANs with other CNNs, such as Xception, Inception v3, etc. One example is [52], which presents an ensemble strategy of group decision for an accurate diagnosis.

## 5. Current Trends in Designing Skin Lesions Diagnosis Systems

**Figure 23:**
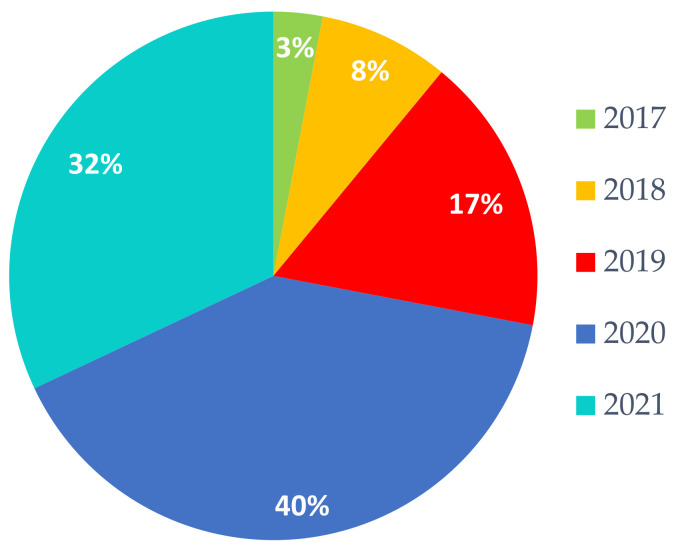
Percent of research papers per year with the highest impact for the new trends in Me detection by NN.

As we mentioned earlier SL and, especially, Me are frequent and dangerous diseases. Simple and early detection might represent an important aspect for treating Me. That is why researchers are still looking for new, more effective methods for the early detection of melanoma. Therefore, this study tries to concentrate on the trends in designing systems dedicated to SL/Me detection, segmentation, and classification. Most of the recent papers are based on NNs but there are also other studies based on classic classifiers such as KNN and SVM. In terms of new trends of using NNs for detection, segmentation, and classification of Me, we noticed the following: systems using one single CNN most often modified and adapted for Me, systems using multiple CNNs, and systems using CNN combined with other classifiers. Figure 23 illustrates the percentage of research papers per year, between 2017 and 2021 which had the highest impact in terms of designing future such systems. It can be seen that most of the important papers related to SL/Me detection, segmentation, and classification were released in 2020. The year 2021 is also promising, since, even if it is not over, with an indexing delay, it captured our attention in terms of importance.

It can be observed the fact that almost all important CNNs were learned with TL techniques using different DSs already mentioned in previous chapters. There were also some studies in which the authors designed their own CNNs or modified existing ones. Our review shows the fact that researchers in this domain were interested in almost all important CNNs including the state-of-the-art: AlexNet, GoogLeNet, VGG, ResNet, Xception, U-Net, DenseNet, MobileNet, YOLO, different types of GANs, and others.

The trend is that researchers, similar to the case of other domains, experimented with different small CNNs and then transited to more complex and deep ones; for example, a transition to ResNet and to other networks using residual connections to improve performance-related indexes.

As we already mentioned, such systems are designed using:

- One CNN, most often modified and using TL technique;
- Multiple CNNs (combined CNN by data fusion into a global classifier);
- One or multiple CNNs combined with other classifiers;
- Other techniques/classifiers.

### 5.1. Melanoma Detection Using One Convolutional Neural Network

Many papers present Me and other SL detection systems designed using only one CNN. Most of them studied different CNNs to compare the obtained results and then to select the one matching the best performances. For instance, in [45] a DL-based approach for SL classification via different individual CNN architectures such as AlexNet, GoogLeNet, VGG, and ResNet, on ISIC 2017 DS is illustrated.

An example of a support system based on NN to help physicians improve their results in categorizing the seven most common pigmented SLs is described in [47]. The paper compares eight deep NNs (VGG16, VGG 19, ResNet 34, ResNet 50, SEResNet 50 (Squeeze-and-Excitation ResNet 50), ResNet 101, EfficientNet B5, and MobileNet) in different training conditions, using images randomly taken from ISIC and HAM1000 DSs. The authors in [113] proposed a method for automated Me detection and segmentation using a modified deep regional convolutional NN to reduce the investigation area and the fuzzy C means algorithm for precise segmentation. The dermoscopic images were from ISIC 2016 DS.

Existing networks modified to achieve a more accurate one are presented in [114]. The authors proposed a modified U-Net version. This new structure took advantage by combining DenseNet and ResNet to improve the performance of U-Net in SL segmentation. The convolutional layers of the encoder are intercalated with context modules containing dense connections. These modules are residual blocks. Similarly, the up-sampling layers of the decoder are intercalated with Localize modules. The new skip connection between the decoder and encoder is named the Dense Skip Connection.

A new trend in designing such systems with more accurate results in SL detection is represented by 3D CNN. For example, the authors in [115] proposed a 3D fully CNN named Hyper-net to achieve a more accurate segmentation of Me from hyperspectral pathology images. The hyperspectral images, as input for Hyper-net, are represented by cubes of size 256 × 256 × 16. The authors combine the dilated convolution for multi-scale features with the standard convolution. Between encoder and decoder blocks there is a fusion path. The output of the decoder is a 3D cube with the same size as the input 3D cube. To enhance the training efficiency, residual learning was inspired by V-net [116].

**Figure 24:**
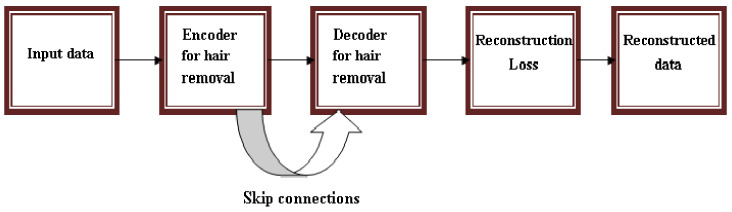
The schematic architecture of the proposed system for hair removal from skin lesion images from [117].

Another new direction in the use of NN for Me detection is the preprocessing tasks. An example of such a network can be seen in [117], a recently published paper that proposed an encoder–decoder CNN for hair removal (Figure 24).

### 5.2. Melanoma Detection Using Multiple Convolutional Neural Networks (Combined)

Combining multiple networks into a complex system can lead to improved SL detection and classification performances. From this point of view, we distinguish two tendencies: (i) the use of several networks, separately, for different functions (detection, segmentation, and classification), either in cascade or in parallel, and (ii) the use of several networks for the same function and the combination of individual decisions, by fusion, for the final decision. For instance, the authors in [50] provided a solution for precise SL analysis by proposing a multi-task DL framework based on a Feature Pyramid Network (FPN), Region Proposal Network (RPN), and three subnets (for classification, detection, and segmentation). The subnets are fed with the outcomes from FPN and RPN (determining the RoI) and they run in parallel to obtain a combined and more precise result for skin lesions analysis and prediction. The framework is based on the design of a loss function based on focal loss (RPN loss function) and the Jaccard distance to solve the SL classes imbalance issue for the image DS. The ISIC 2016 and ISIC 2017 challenge DS were used.

The new diagnosis system presented in [38] is a solution for Me detection based on DL techniques. The system contains two main modules: (RoI detection using Mask R_CNN and RoI classification, using TL for ResNet152 which was previously trained with ImageNet DB).

**Figure 25:**
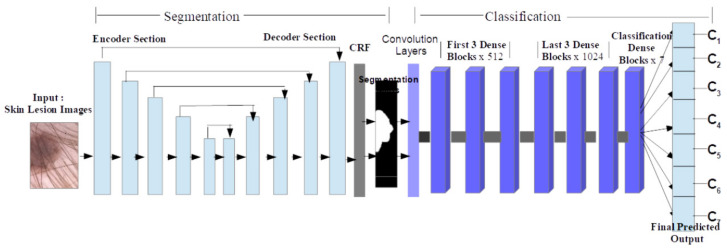
The architecture of the proposed system for skin lesion classification [1].

Two CNNs are also combined in [1] to perform an accurate classification (95% ACC) of SL. As can be seen in Figure 25, the image containing an SL flows through the first CNN (encoder–decoder type) designed for segmentation purposes, and afterwards, the segmented SL is considered as the input in the next CNN composed of merged dense blocks, for classification. The experiments were conducted using the HAM10000 DS.

A similar system design was proposed in [41], a recent paper proposing a combination of two modules: a segmentation module used as a pre-requisite and a classification module. The difference here is that, first, the authors experimented with multiple DSs such as ISIC 2016, ISIC 2017, and ISIC 2018, and second, multiple CNNs were involved and analyses were conducted for each of them. The SL segmentation is performed by FrCN and the classification is performed by the following NN: Inception v3, ResNet 50, Inception-ResNet v2, and DenseNet 201. Case studies of two, three, and seven classes are considered.

The authors in [42] proposed a generalized architecture for multi-class classification of skin cancer. This paper covers five convolutional NN architectures for different experiments, such as: Xception, NASNet-Large, Inception-RestNet v2, Inception v3, ResNeXt101 and the ensembles: Inception-ResNet v2 + Xception, Inception v3 + Xception, Inception-ResNet v2 + ResNetXt 101 + Xception and Inception-ResNet v2 + ResNetXt 101. Experiments were performed using the HAM10000 DS and the best ACC was obtained for ResNetXt 101 + Inception-ResNet v2 (92.83%).

**Figure 26:**
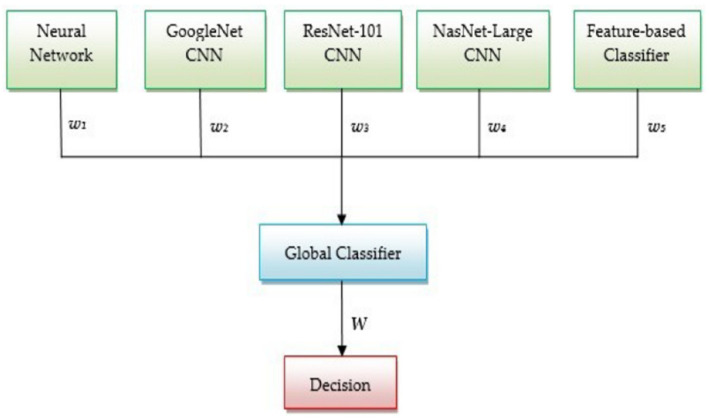
Multi-network system architecture based on decision fusion for Me detection [5].

Creating a complex multi-network system based on the fusion of decisions of individual NNs with the help of a final NN can increase the detection performance of Me. For example, the authors in [5] proposed a Me detection system characterized by the following new aspects: (a) use of multiple CNN as individual classifiers, (b) use of a hybrid structure which makes a decision fusion between four CNN-based classifiers and a classifier based on texture features, (c) use of another CNN considered as a global classifier having as input the probabilities of individual classifiers (considered as weights). The CNNs used were: a custom NN, GoogLeNet, ResNet-101, NasNet-Large, and a Perceptron (Figure 26).

Similar to the authors of [5], the authors in [45] proposed an ensemble of CNNs (GoogLeNet, AlexNet, ResNet, and VGGNet) for SL classification based on the CNN output interpretation, demonstrating that it is a meaningful approach.

**Figure 27:**
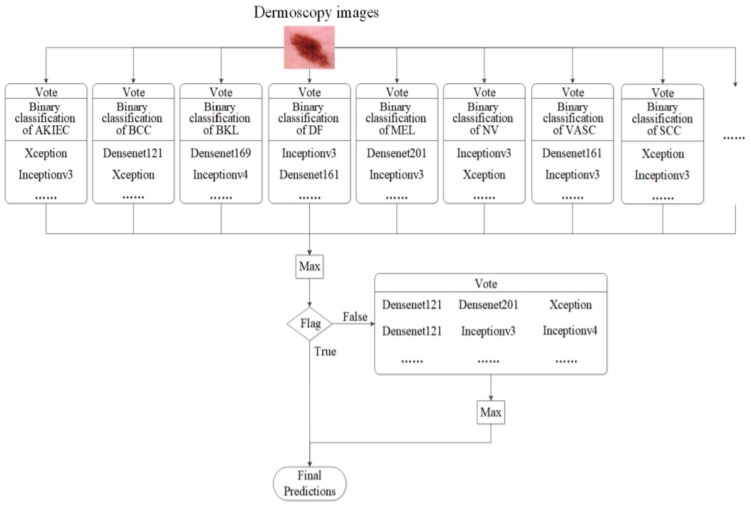
Ensemble strategy of the group decision [52].

A complex system for Me and SL diagnosis based on multi-CNN and a voting scheme is proposed in [52]. As seen in Figure 27, the classification system is based on multiple sub-modules, each of them voting a single dermoscopic image and providing a value. The maximum value is then compared with a threshold. In case it is smaller than the threshold, the classification will be performed by a group decision conducted by a large module (Vote) composed of other CNNs. Thus, a final, more accurate decision related to the classification is obtained.

**Figure 28:**
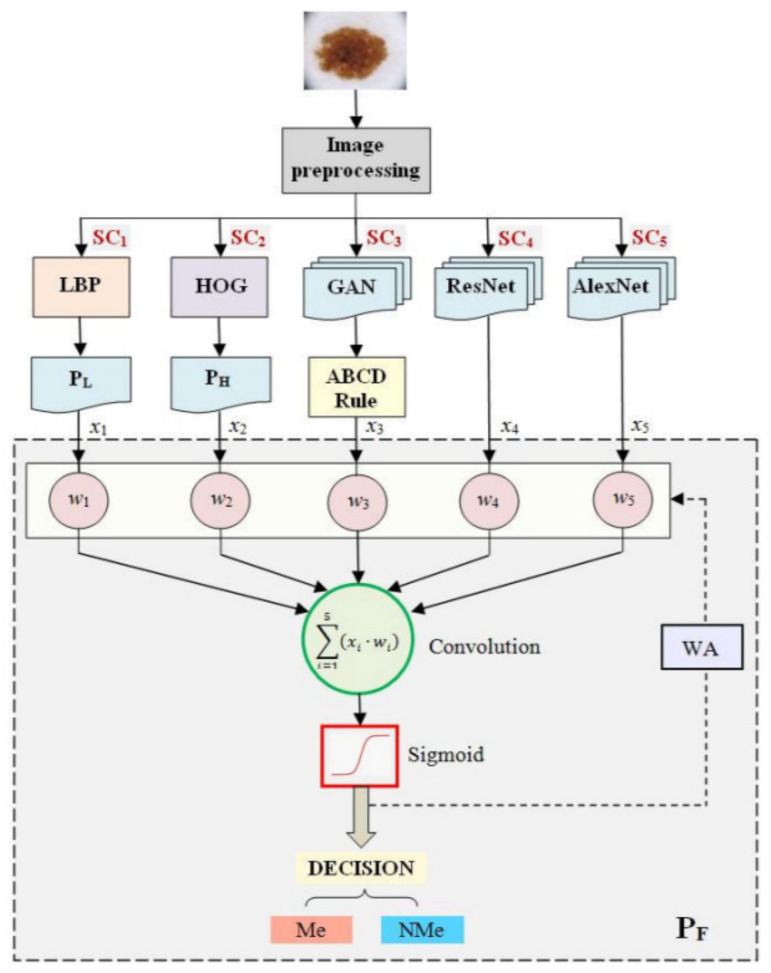
The architecture of the Me classification system proposed in [6], based on several NNs connected on two levels of classification.

Through the appropriate use of several NNs, it is possible to move from subjective classification decisions of individual networks to a decision considered more objective of the global classifier also represented by an NN [6]. In this specific research paper, as can be seen in Figure 28, the authors proposed a system based on a decision taken from multiple NNs. This system is based on six classifiers (NN-based) connected to two operational levels. The first level contains five subjective (individual) classifiers, while in the second level, there is a Perceptron-type classifier that decides whether the final decision is a Me or not. The final decision is based on the learning-adjusted weights from the first level. In the learning phase, a weight is assigned to each subjective classifier, according to the classification accuracy. In the testing phase, the outputs of these classifiers are the probabilities offered. The convolution law of the final classifier is made up of the weights and probabilities of the subjective classifiers. It is considered that the final classifier is an objective one. The subjective classifiers are the following: (a) two NN namely ResNet 101 and AlexNet, (b) two perceptrons having as inputs LBP histogram and HOG, respectively, and (c) an ABCD-based classifier with a GAN for primary segmentation.

### 5.3. Systems Designed Using Convolutional Neural Networks Combined with Other Classifiers/Techniques

As we mentioned earlier, there are SL diagnosis systems designed using CNNs combined with other techniques/classifiers to obtain better results in terms of statistic performance-related indexes. An example of this is [118], which aims to provide two solutions (benign/malignant) for a precise and optimum classification of SL by proposing two corresponding systems. The architecture of both systems specifies a lesion segmentation block based on HLPSO (Hybrid Learning Particle Swarm Optimization) and modified K-means as a common initial block. The first system is then composed of other two blocks: feature selection block (based on HLPSO, KIRSCH, and SLBP) and SL classification block (based on KNN and SVM). The second system is an adaptive one, based on evolving DCNN (CNN driven by HLPSO for parameters/hyper-parameters optimizations).

Another example of such a diagnosis system is [90], which aims to provide a solution for the SL detection and segmentation system based on YOLO v3 for SL detection and GrabCut algorithm for accurate segmentation. YOLO v3 was chosen for the detection part since it already proved to be much faster and has better precision and accuracy in detection than other methods such as RCNN (region-based convolutional neural network)/Fast-RCNN/Faster-RCNN. The system being proposed also contains a preprocessing module able to process the image (e.g., hair removal) before the SL detection phase and the segmentation phase.

**Figure 29:**
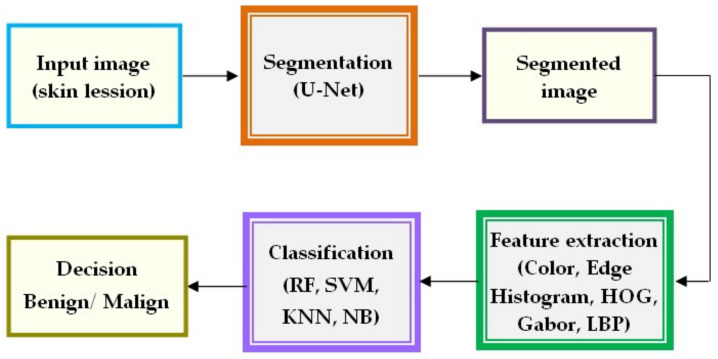
The schematic architecture of the skin lesion classification system based on CNN for the segmentation, feature extraction, and intelligent classification [59].

The authors in [119] aim to provide a solution for designing a Me classification system based on CNN and a custom new regularizer for controlling the complexity of the classifier and thus making it more accurate. The results are indeed more accurate and precise when compared with other works from the existing literature. Similarly, another research paper [59] proposed a Me diagnosis system based on a combination between CNN and intelligent classifiers based on texture features. As seen in Figure 29, this paper presents an architecture based on a segmentation block using U-Net, a feature extraction block using a color feature, HOG, LBP, and a classification block using RF (random forest), SVM, KNN, and NB (Naive Bayes).

**Figure 30:**
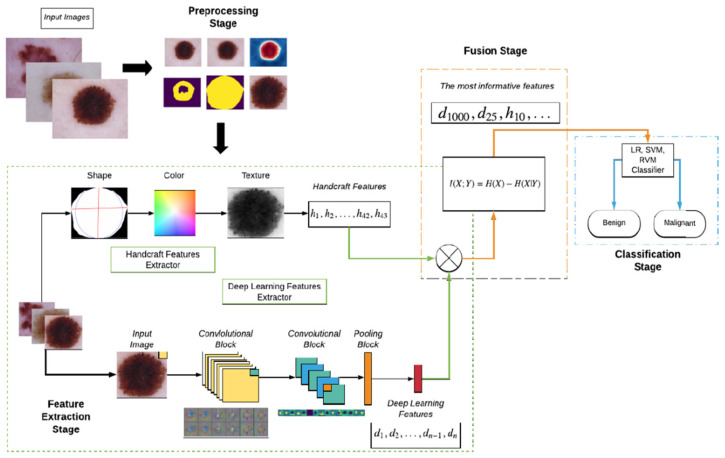
The architecture of the SL classification system proposed in [43].

The authors in [43] proposed a new solution for SL classification system based on handcrafted features (color, texture, etc.) fused with features learned by TL on pre-trained CNNs such as VGG 16, VGG 19, MobileNet, ResNet 50, Inception v3, Xception, DenseNet 201, MobileNet v1, and MobileNet v2. The fusion block identifies the most important features and passes them to the classification block which is based on Linear Regression, SVM, and a Relevant Vector Machine. Experiments on the system proposed in Figure 30 were conducted on ISIC 2018 DS and performance results were analyzed for each mentioned CNN. The best results were obtained by the one using MobileNet v2 (about 90% ACC in the testing phase).

In terms of Me detection and segmentation systems, a recent important paper is considered [88], where YOLO v4 was used for Me detection and an Active Contour Segmentation approach for Me segmentation. As DSs, ISIC 2016 and 2018 were used. It can be seen that recent papers aim to use real-time object detection methods such as YOLO v3 and YOLO v4 to achieve Me detection.

A synthesis of the characteristics of the most important papers regarding the trends of using NN in Me and SL detection is presented in Table 4.

### 5.4. Systems Designed Using Other Techniques

Our study of course identified Me and other skin lesion diagnosis systems using other techniques apart from convolutional NNs. An example of such a paper would be [33], which aims to provide a solution for designing an SL segmentation system based on the Artificial Bee Colony algorithm for obtaining an optimum threshold value for Me detection for the segmentation phase. From an architecture point of view, the system is composed of three modules: the preprocessing (applying median filter), the application of the ABC algorithm for finding the optimum threshold value to be used for Me detection, and the segmentation module.

Another example of such a paper is [120], which tries to provide a solution to the problem of having multiple small skin lesion DSs (less training data towards the classification of Me) by introducing a TL framework called TrCSVM (Transfer Constituent Support Vector Machine) which can transfer knowledge retrieved from a source training set to multiple target training sets, thus obtaining a more efficient classification model capable of classifying various SLs. This framework is based on FBDA (Feature-Based Domain Adaptation) and uses SVM and TrAdaBoost (Transfer AdaBoost).

## 6. Discussion

The paper presented the most used techniques based on NN for detection, classification, and segmentation of SL and, especially, Me. The focus was on new trends in such applications. To this end, we analyzed 134 references, most of them from the period 2017–2021. The most performant new systems for Me detection contain multiple DCNNs selected on a performance criterion and grouped either with each other, based on the fusion of decision, or with other classifiers based on texture, shape, and color features. In this way, we move from subjective classifications, specific to individual classifiers (NN), to a more objective classification, that of the global classifier. This classifier considers, according to pre-established criteria, the decisions of the subjective classifiers, but makes its own decision. The individual classifiers should be chosen so that the objective classifier can compensate for possible individual classification errors. Another interesting combination of NN would be the pipeline type, based on jobs; for example, the first network performs primary processing, the second segmentation, and the third classification. There were also implementations of new networks based on the introduction in the structure of a known network, as intermediate modules, smaller networks. The performances obtained depend both on the proposed network solution and on the DS used (including the selected images).

The vast majority of analyzed papers were selected from Web of Science as the most trusted publisher global citation DB. Searches were focused on the following criteria: (a) topics as Me and NNs, (b) new trends (papers between 2017 and 2021), (c) the number of citations, (d) impact factors for journals, and (e) rate of ISI indexing for proceedings papers. We identified eight review or survey papers between 2018 and 2021 [134,135,136,137,138,139,140,141]. Table 5 highlights the characteristics of these articles and the differences of our article, marked as positive aspects or contributions.

As already mentioned above, over the years, numerous studies have been conducted on this topic. In 2009, Fernandez Alcon et al. [18] analyzed the SL pigment and performed Me diagnosis with an automatic imaging system proposed by the authors. The detection ACC was improved by combining the classification results with information such as gender, skin type, or the age of the patient. First, the segmentation, background correction, and threshold-based segmentation are analyzed. Then, the ABCD-based method is used to complete the feature extraction step. In the end, pattern recognition is used to perform the classification in Me and non-Me lesions. From the Dermnet dataset (DS), 152 images were used to evaluate the system, from which 107 were Me images and 45 benign SL images. The ACC given by the system was 86% [18].

In 2011, Capdehourat, G. et al. [19] proposed an ML-based approach that classifies SL as malignant or benign. In the preprocessing step, the authors used the already well-known Dullrazor algorithm, developed by Lee, T. et al. [142] to remove the hair present in the lesion. In the segmentation step, the Otsu method, which performs automatic image thresholding, was used with the specification from the authors that this method failed in certain pathological cases. Texture, color, and geometrical features were extracted in the feature extraction step. For the classification step, AdaBoost with C4.5 decision trees was used. According to the authors, this system performance was analyzed by calculating the specificity (77%) and sensitivity (90%). Two years later, Razmjooy, N. [143] et al. proposed another ML-based system that helps with Me detection. In the preprocessing step, a new algorithm for hair removal is used, other than Dullrazor. As specified by the authors, the hair removal algorithm consists first in applying canny edge detection. Then, a thicken operation, dilatation operation, and addition to the original image are used. The segmentation step is based on morphological operations while, for the feature extraction step, new features, based on asymmetry and irregular border quantification, are applied. An ACC of 95% was given by the Support Vector Machine (SVM) used as a classifier.

The authors in [20] developed a system based on the same classical method. They preprocessed the images by applying noise removal techniques and used the threshold-based method for image segmentation. ABCD rule and Principal Component Analysis (PCA) are then used to extract the features. The classification made with the help of SVM showed an ACC of 82.2%, a specificity of 86.93%, and a sensitivity of 77%. The evaluation of the system was made on 282 images (133 Me images and 149 benign images) selected from several DSs such as Dermquest, Dermnet, and Dermis.

Starting with 2015, the attention of most researchers turned to DL methods. Codella N. et al. [144] combined the SVM algorithm with sparse coding and DL techniques to develop a system that reaches a 93.1% ACC in the case of an Me class, an atypical class, and a benign class. As for feature extraction, a pre-trained Caffe CNN (Convolutional Neural Network) was used. The performance evaluation of the system was conducted on the International Skin Imaging Collaboration (ISIC) DS.

The authors in [145], proposed a system based on two main steps: preprocessing and classification. For the classification, a pre-trained CNN that contains two convolutional layers was used. The system showed an 81% ACC on Me detection.

Pomponiu V. et al. [146], just like in [144], developed an SL detection system that used a CNN as a feature extractor showing an ACC of 93.64%, a specificity of 95.18%, and a sensitivity of 92.1%. After the data augmentation was conducted, an AlexNet pre-trained CNN was used to extract the features and a K-nearest neighbor (KNN) algorithm was used for classification.

An NN ensemble method for Me detection was proposed by Xie F. et al. [147] in 2016. This paper presents a system that primarily has three steps. The first step is the segmentation step that is conducted with the help of a self-generating NN. The second step is the feature extraction step using PCA, followed by the classification step that is performed by using the NN ensemble method. The proposed classifier refers to a combination of Fuzzy NN and backpropagation NN. The performance evaluation on two different DSs provided by a local hospital showed an ACC of 94.17% and a sensitivity of 95%.

Some authors, such as Attia M. et al. [148] have only addressed the subject of Me segmentation. The authors used CNN and Recurrent NNs (RNN) to develop a high ACC of Me segmentation system. The proposed architecture contains seven convolutional layers that represent the autoencoder part and four recurrent layers. A total of 900 images obtained from ISBI 2016 challenge [14] were used for evaluating the algorithm. A Jaccard Index of 93% and segmentation ACC of 98% were obtained.

Li Y. et al. [149] also used DL techniques to detect Me. Two fully CNNs, named the Lesion Feature Network and Lesion Index Network, were used to complete the feature extraction, segmentation, and classification steps. Calculation of the distance heat map is then conducted to improve the detection. For the feature extraction step, a straightforward CNN (Lesion Index Network) was used. The evaluation was performed on ISIC 2017 DS [25]. In the case of the Lesion Index Network, the obtained ACC for image classification and segmentation was 91.2% while the obtained Jaccard index was 75.3%. Regarding the Lesion Feature Network, the performance was evaluated in terms of sensitivity and precision (69.3% and 42.2%) [149].

A study for CNN optimization study was performed by Zhang L. et al. [35] in 2019. The purpose of this study was to improve the training of network weights and biases by applying a meta-heuristic procedure. To minimize the learning error, the authors proposed the whale optimization algorithm. To evaluate the system ACC (91%), Dermrequest and Dermis DSs were used.

DL techniques were also used by Milton [25], where the following NNs: SENet154, InceptionResNetV2, PNASNet-5-Large, and InceptionV4 were applied, tested, and compared. The third mentioned one showed the best results (76% validation score) when applied on ISIC 2018 DS [25,28].

A more complex system configuration containing a global decision system that integrates the most commonly used DL methods was proposed in [5]. So, an NN-based method, three CNN-based methods, including NasNet-Large, GoogLeNet, and ResNet-101, and a classical ML-based method were combined to set the fusion weights. The system was evaluated on PH2 [22] and ISIC 2019 [25,28,150] DSs. The best ACC was obtained on PH2 DS (95%), while the ACC obtained on ISIC 2019 was 93%.

## 7. Conclusions

Neural networks as part of AI algorithms are increasingly being researched in imaging applications as a support system for diagnosing SL and detecting Me. New DBs and even challenges regarding the classification of SL are constantly appearing. That is why there is interest in improving these classifiers for detecting and tracking the evolution of SL even from a distance, with great accuracy. The best results were obtained using multiple NNs for different functions and decision fusion. Observing the tendency for increasing use of neural networks in detecting Me, we can say that this area of interest and the manner of solving problems are objectives of great interest in the integration of artificial intelligence in medicine. The use of NN in the detection of melanoma may be involved in a support system for the dermatologist who ultimately has to decide to either indicate a biopsy if at least one of the dermatologist’s diagnoses and the support system (a helpful method) indicates Me or to investigate if there is another type of cancerous lesion. In the latter case, the system can be taught to detect other types of malignant SLs. However, the system cannot make final decisions on its own. Given the evolutionary trends of neural networks, it is expected that such systems will increase their performance by using improved, adapted, and combined networks. A future direction to follow is the use of these systems to detect Me that develops under the nails, which is currently a more complicated case of diagnosis. We do not know of such an algorithm and we have not found it in the literature. If the nail is still transparent, an image enhancement algorithm can be used to separate the Me from the nail. If the Me has attacked the nail, the network must be learned with the nail.

## Figures and Tables

**Table 1:** Performance indicators used in the review.

Indicator	Formula	Indicator	Formula
Accuracy	$\frac{\mathrm{TP}+\mathrm{TN}}{\mathrm{TP}+\mathrm{TN}+\mathrm{FP}+\mathrm{FN}}$	Sensitivity	$\frac{\mathrm{TP}}{\mathrm{TP}+\mathrm{FN}}$
Precision	$\frac{\mathrm{TP}}{\mathrm{TP}+\mathrm{FP}}$	Specificity	$\frac{\mathrm{TN}}{\mathrm{TN}+\mathrm{FP}}$
Dice Coefficient	$\frac{2∙\mathrm{TP}}{2∙\mathrm{TP}+\mathrm{FP}+\mathrm{FN}}$	Jaccard index	$\frac{\mathrm{TP}}{\mathrm{TP}+\mathrm{FN}+\mathrm{FP}}$

**Table 2:** Skin lesions DSs frequently used in Me detection.

DS Name	Reference	Availability	SL	Me
PH2	[22]	Publicly available	200	40
ISIC 2016	[14]	Publicly available	900	273
ISIC 2017	[25]	Publicly available	2000	374
ISIC 2018, HAM10000	[25,28]	Publicly available	10,015	1113
ISIC 2019	[23,26,36]	Publicly available	25,333	4522
ISIC 2020	[23]	Publicly available	33,126	584
DERMQUEST	[37]	Publicly available	126	66
MED-NODE	[29]	Publicly available	170	100
DERMNET	[31]	Publicly available	22,500	635
DERMIS	[33,34]	Publicly available	397	146
DERMOFIT	[30]	Purchase only	1300	76

**Table 3:** Family of NNs used for Me diagnosis used in references.

NN family	Representatives	References
ResNet	ResNet 34, ResNet 50, SEResNet 50, ResNet 101, ResNet 152, FCRN	[5,6,31,38,39,40,41,42,43,44,45,46,47,48,49,50]
Inception/GoogLeNet	GoogLeNet (Inception v2), InceptionResNet-v2, Inception v3, Inception v4	[5,36,40,41,42,43,45,46,49,50,51,52]
U-Net	U-Net	[43,49,53,54,55,56,57,58,59,60,61,62,63]
GAN	GAN, SPGGAN, DCGAN, DDGAN, LAPGAN, PGAN	[6,52,56,64,65,66,67,68,69,70,71]
DenseNet	DenseNet 121, DenseNet 161, DenseNet 169, DenseNet 201	[1,31,40,41,49,50,52,67,71,72]
AlexNet	AlexNet	[6,12,45,46,73,74,75,76]
Xception	Xception	[40,42,43,46,49,52,67]
EfficientNet	EfficientNet, EfficientNetB5, EfficientNetB6	[47,77,78,79,80,81,82,83]
VGG	VGG 16, VGG 19	[40,43,45,46,47,54,84,85]
NASNet	NASNet, NASNet-Large	[5,31,42,86]
MobileNet	MobileNet, MobileNet2	[40,43,47,87]
YOLO	YOLO v3, YOLO v4, YOLO v5	[88,89,90]
FrNet	FrNet	[91]
Mask R_CNN	Mask R_CNN	[92]

**Table 4:** Synthesis of the most important papers regarding the trends of using NN in Me and SL detection.

Ref/ Year	Goal/Novelty	Description	NN Type/Function	Data Set	Me or SL + Me	Data Aug.	Performance Indicators (%)		
							ACC	F1	IoU
[45]/ 2018	DL-based approach for SL classification via the fusion of different individual CNN architectures.	Ensemble of CNNs with different fusion-based methods and selection of the best performing one.	GoogLeNet, Alexnet, ResNet, VGGNet/ classification	ISIC 2017	SL + Me	Yes	90.30	NA	NA
[90]/ 2019	Pipeline architecture for SL segmentation, combining YOLO v3 and the GrabCut algorithm.	Combining YOLO v3 and the GrabCut Algorithm for SL segmentation.	YOLOv3/ detection and segmentation	PH2, ISIC 2017	SL + Me	NA	92.99 to 97.00	84.26 to 88.13	74.81 to 79.54
[113]/ 2019	A DL method is proposed for automated Me detection and segmentation using dermoscopic images.	Skin refinement, localization of Me region, and, finally, segmentation of Me (fuzzy C means).	Deep region-CNN/detection and segmentation	ISIC 2016	Me	NA	94.80	95.89	93.00
[121]/ 2019	New FCNN architecture for SL segmentation—DermoNet.	FCNN contains densely connected convolutional blocks and skip connections.	FCNN—DermoNet/ segmentation	PH2, ISIC 2016, ISIC 2017	SL + Me	Yes	NA	89.40 to91.50	82.50 to 85.30
[53]/ 2019	Model enhanced by employing a multi-stage segmentation approach.	FCNN based on U-Net with batch normalization.	FCNN/ segmentation	ISIC 2018	SL + Me	Yes	NA	90.00	83.00
[122]/ 2019	Encoder–decoder structure with an intermediate module (attention module).	The architecture contains three modules: the encoder that extracts features from a raw image; the decoder that generates the SL classes; the attention module for guiding the decoder to attend at different locations.	Encoder–Decoder	ISIC 2017	SL + Me	NA	72.3	NA	NA
[39]/ 2020	New deep CNN-based model for face skin disease classification using a triplet loss function.	Fine-tuning layers of ResNet152 and InceptionResNet-v2.	ResNet152, Inception ResNet-v2/classification	From a hospital in Wuhan China	SL + Me	NA	87.42	NA	NA
[123]/ 2020	A new method called a “Lesion classifier” is derived from pixel-wise classification.	Encoder–Decoder Network Connected through skip pathways. Softmax modules for output.	Encoder–Decoder/ detection and segmentation	ISIC 2017, PH2	Me	Yes	95.00	92.00	NA
[124]/ 2020	New skin image classification method using multi-tree genetic programming.	Various local and global features are extracted from skin cancer images. The classification method uses genetic programming.	NA/ classification	PH2, Dermofit	SL + Me	NA	96.42 to 80.64	NA	NA
[88]/ 2020	New scheme for Me localization and segmentation using YOLOv4 and active contour segmentation. Detecting multiple Me presented in a single image.	The skin refinement step removes the unnecessary artifacts automatically. A framework consisting of three phases: skin enhancement, Me localization, and Me segmentation.	YOLO v4/ detection and segmentation	ISIC 2016, ISIC 2018	SL + Me	Yes	94.00	92.00	96
[41]/ 2020	DL-based CAD system with precise SL boundary segmentation and accurate classification for clinical diagnosis of SL	Cascaded full resolution CNN for segmentation and Inception-v3, ResNet-50, Inception-ResNet-v2, and DenseNet-201 for classification.	DCNN/ segmentation and classification	ISIC 2016, ISIC 2017, ISIC 2018	SL + Me	Yes	87.74 to89.28	77.84 to 81.28	NA
[125]/ 2020	Me detection using an optimized set of Gabor-based features and a fast MNN classifier.	Gabor features combined with a fast (Multi-Level Neural Network) MNN.	MNN/ classification	PH2	Me	NA	97.50	NA	NA
[89]/ 2020	YOLO v3 algorithm combining with two-phase segmentation based on the graph theory using minimal spanning tree concept and L-type fuzzy-based approximations.	YOLO v3 for Me detection and segmentation based on graph theory.	YOLOv3/ detection and segmentation	PH2, ISIC 2017, ISIC 2019	Me	NA	93.38–97.50	87.89–93.97	79.84–88.64
[43]/ 2020	Fusing method that employs relevant mutual information obtained from handcraft and DL features obtained from DCNN.	ABCD rule combining with DCNN features employing mutual information measurements.	VGG-16, VGG-19, MobileNet v1, ResNet-50, Inception v3, Xception, DenseNet-201/ classification	HAM10000	SL + Me	Yes	92.40	90.00	NA
[5]/ 2020	Integration of different NNs into a global fusion-based decision system. For the fusion weights, there are used the results, obtained by each NN.	A global classifier is implemented considering individual classifiers as the proposed NNs. The global classifier used partial decision fusion.	CNN, GoogLeNet, ResNet101, NasNet-Large, Perceptron/ classification	PH2, ISIC 2019	SL + Me	Yes	88.33 to 93.33	86.79 to 92.31	NA
[126]/ 2020	Optimal CNN to predict skin cancer.	A new technique of using an improved whale optimization algorithm for optimizing the structure of CNN for skin cancer detection.	Optimized CNN/ detection	Dermquest, DermIS	SL + Me	NA	95	NA	NA
[6]/ 2020	An objective classifier containing five subjective classifiers (two texture-based classifiers with perceptrons and three NNs end-to-end type) for Me detection.	A multi-NN-based system containing six NNs and feature extraction algorithms. The final classifier is also an NN.	Perceptrons coupled with feature extraction, GAN, ResNet, AlexNet/ segmentation, and classification	PH2, ISIC 2019	Me	Yes	97.50	97.40	NA
[47]/ 2020	Establishing how DL frameworks trained in large DSs can help non-dermatologists improve their performance in categorizing pigmented SL.	The performances of eight DCNNs are compared in different training conditions.	VGG16, VGG19, ResNet34, 50, 101 SEResNet50, EfficientNetB5, MobileNet/ classification	HAM10000	SL + Me	NA	75.73 to 84.73	NA	NA
[127]/ 2020	New CNN architecture for SL segmentation, with an attention mechanism and high-resolution feature maps.	Proposed CNN with K consecutive HRFB (high-resolution feature block) for SL segmentation with more accurate SL boundaries.	CNN with HRFB/ segmentation	PH2, ISIC 2016, ISIC 2017	SL + Me	Yes	93.80 to 94.90	86.20 to91.90	78.30 to 85.80
[58]/ 2020	Improved U-Net for SL segmentation.	The architecture is proposed with a modified U-Net, in which a bilinear interpolation method is used for up-sampling with a block of convolution layers followed by parametric ReLU.	U-net/ segmentation	NA	SL + Me	Yes	94.00	88.00	NA
[128]/ 2020	A variant of the particle swarm optimization algorithm, HLPSO, for SL segmentation and classification.	Combining HLPSO with DCNN and a K-Means clustering algorithm.	DCNN/ classification and segmentation	ISIC 2017	SL + Me	NA	91.37	NA	73.15
[118]/ 2020	Global-Part CNN, considering the local information and global information with equal importance.	Ensemble of two CNNs for local and global information, based on data fusion.	Ensemble of two CNN/ classification	ISIC 2016, ISIC 2017	SL + Me	Yes	85.70 to 92.50	NA	NA
[24]/ 2021	New model, ASCU-Net (Attention Gate, Spatial and Channel Attention U-Net) using convolutional block attention modules for SL segmentation.	Due to the attention module, ASCU-Net accelerates the learning phase.	ASCU-Net based on U-Net and triple attention mechanism/ segmentation	PH2, ISIC 2016, ISIC 2017	SL + Me	Yes	95.40	90.80	84.50
[129]/ 2021	Design of a new DCNN model with multiple filter sizes—Classification of Skin Lesions Network (CSLNet).	Fewer filters, parameters, and layers to improve SL classification performances.	DCNN (CSLNet)/ classification	ISIC 2017, ISCI 2018, ISIC 2019	SL + Me	Yes	89.58 to93.25	89.75 to 93.47	81.50 to 88.20
[79]/ 2021	New NN based on Efficient-B5.	A deeper, wider and higher resolution NN for Me classification based on fine-grained feature representations.	Efficient-B5/ classification	ISIC 2020	Me	NA	NA	NA	NA
[130]/ 2021	Testing different NN for recognition of pigmented SL	Testing different NN for recognition of pigmented SL	ResNet50, DenseNet121, VGG16/ classification	ISIC, HAM10000,PH2, BCN20000, SKINL2	SL + Me	Yes	NA	NA	NA
[131]/ 2021	An extensive analysis of twelve CNN architectures and eleven public images DBs.	An extensive analysis of twelve CNN architectures and eleven public image DBs for automatic Me automatic diagnosis.	DenseNet121, 169, 201, Inceptionv3, v4, ResNet50, InceptionResNet v2, Xception, VGG16, 19, Mo-bileNet, and NASNetMobile/detection	PH2, ISIC 2016, ISIC 2017, HAM10000, MED-NODE, MSK1, 2, 3, 4, UDA 1, 2.	Me	Yes	NA	NA	NA
[87]/ 2021	Combining the MobileNetV2 with the Spiking Neural Network (SNN) into a DCNN for the classification.	Three NNs connected into an intelligent decision support system for skin cancer detection.	Autoencoder, MobileNetv2, SNN/ classification	ISIC	Me	Yes	95.27	NA	NA
[132]/ 2021	New and efficient adaptive dual attention module (ADAM) for automated skin lesion segmentation.	The proposed ADAM modules are integrated into a dual encoder architecture.	Dual encoder + ADAM/ segmentation	ISIC 2017, ISIC 2018	SL + Me	Yes	96.36	91.63	84.70
[133]/ 2021	New Siamese NN and architecture named Tensorial Regression Process to detect SL evolution.	A pair of SL images are compared to detect the possible evolution of SL to Me. To this end, a segmentation loss is incorporated into NN as a regularization term.	Siamese NN/ detection and segmentation	Sydney Melanoma Diagnostic Centre	SL + Me	NA	74.10	NA	NA
[71]/ 2021	SL augmentation DS by StyleGAN and DenseNet201 for classification.	Two NNs are used to improve SL classification: a special GAN for data augmentation and DenseNet 201 for classification with a special strategy of TL	GAN (StyleGAN). DenseNet201/ classification	ISIC 2018, ISIC 2019	SL + Me	Yes	93.64	NA	NA

**Table 5:** Recent review/survey papers on similar topics.

Paper/Year	Description	Period	No. of References	Our Differences
[134]/2018	A critical and analytical survey of different algorithms for performing segmentation of SL.	2007–2018	29	New period (2017–2021). Focused on Me and NNs. More references. Focused on new trends (including 2021).
[135]/2018	Medical (general) image segmentation and classification using CNN.	2010–2018	96	New period (2017–2021). Focused on Me and NNs. More references.
[136]/2018	SL classification using CNNs.	2012–2018	33	New period (2017–2021). Focused on Me and NNs. More references. Focused on new trends (including 2021).
[137]/2019	Different methods for cancer detection including skin cancers: classical methods (ABCD, different features) and NNs.	1993–2019	167	A modern approach based on ML and NNs. New period (2017–2021). Focused on Me and NNs. Focused on new trends (including 2021).
[138]/2020	Investigating: DBs, Me types, DL techniques, reference sources, and index.	2004–2020	95	Focused on Me and NNs. More references. Focused on new trends (including 2021).
[139]/2020	Survey of the recent architectures of deep CNNs (general). Analysis of CNN’s internal structures.	1982–2020	253	Focused on Me and NNs. Systems of multiple NNs and decision fusion as new trends.
[140]/2021	Methods for detecting skin cancer from SL images.	2011–2020	135	Focused on Me and NNs. More references. Focused on new trends (including 2021).
[141]/2021	A systematic review of DL techniques for the early detection of skin cancer.	1993–2021	82	Focused on Me and NNs. More references. Focused on new trends (including 2021).

## Abbreviations

**Abbreviations**	**Description**
ABCD	Asymmetry Borders-Colors-Dermatoscopic Structures
ACC	Accuracy
CAD	Computer Aided Diagnosis
CNN	Convolutional Neural Network
DB	Database
DCNN	Deep Convolutional Neural Network
DL	Deep Learning
DS	Dataset
F1	Dice Coefficient (F1 Score)
FCN	Fully Convolutional Network
FPN	Feature Pyramid Network
HLPSO	Hybrid Learning Particle Swarm Organization
ILSVRC	ImageNet Large Scale Visual Recognition Challenge
IoU	Intersection-Over-Union, Jaccard Index
ISIC	International Skin Imaging Collaborative
KNN	K-Nearest Neighbor
Me	Melanoma
ML	Machine Learning
MNN	Multi-level Neural Network
NN	Neural Network
PCA	Principal Component Analysis
PSO	Particle Swarm Optimization
RCNN	Deep region based convolutional neural network
ReLU	Rectified Linear Units
RGB	Red-Green-Blue
RPN	Region Proposal Network
SL	Skin lesion
SVM	Support Vector Machine
TL	Transfer learning
YOLO	You Only Look Once
